# Short-term and long-term effects of vitamin D supplementation for preterm infants: a systematic review and meta-analysis

**DOI:** 10.1038/s41372-025-02440-9

**Published:** 2025-10-07

**Authors:** Seung Hyun Shin, Hyun Jung Kim, Ju Sun Heo

**Affiliations:** 1 Department of Pediatrics, Korea University College of Medicine, Seoul, Republic of Korea; 2 Institute for Evidence-Based Medicine, Cochrane Korea, Department of Preventive Medicine, Korea University College of Medicine, Seoul, Republic of Korea; 3 Department of Pediatrics, Seoul National University College of Medicine, Seoul, South Korea; 4 Department of Pediatrics, Seoul National University Children’s Hospital, Seoul, South Korea

**Keywords:** Paediatrics, Outcomes research

## Abstract

Meta-analysis conducted to evaluate the effectiveness of high-dose (≥800 IU/day) and low-dose (<800 IU/day) vitamin D supplementation on preterm infants. Study quality was evaluated using the Revised Cochrane risk-of-bias tool 2 for randomized trials. 21 studies included 1130 infants. Regarding short-term (before 40 weeks’ postmenstrual age [PMA] or at discharge) outcomes, high-dose vitamin D supplementation was associated with increased serum 25-hydroxyvitamin D (25[OH]D) levels (mean difference 15.62 [13.35–17.88]) and growth velocities, as well as decreased vitamin D deficiency (VDD), skeletal hypomineralization, and mortality. In the subgroup analysis of high-dose supplementation stratified by dosage, 800 IU/day significantly increased serum 25(OH)D levels (mean difference 13.99 [9.03–18.95]) and reduced the risk of VDD (risk difference −0.21 [−0.32 to −0.10]) compared to 400 IU/day, without increasing the risk of vitamin D excess. The long-term outcomes assessed after 40 weeks’ PMA or at follow-up visits showed no significant differences in vitamin D status or neurodevelopmental outcomes between the high-dose and low-dose groups. The certainty of the evidence ranges from moderate to very low. High-dose vitamin D supplementation improved short-term outcomes by increasing serum 25(OH)D levels, promoting growth, and reducing mortality. Among the high-dose regimens, 800 IU/day appeared to be the most appropriate dose.

## Introduction

Vitamin D is important for normal bone mineralization. However, preterm infants are vulnerable to vitamin D deficiency (VDD) due to several factors, including decreased transplacental transfer of vitamin D, limited synthesis resulting from prolonged hospitalization, prolonged parenteral nutrition use, and minimal fat mass for the storage of vitamin D and its metabolites [1, 2]. Inadequate vitamin D levels lead to increased risks of metabolic bone disease or rickets in preterm infants [3]. In addition, VDD may be associated with lung maturation, respiratory distress syndrome (RDS), bronchopulmonary dysplasia (BPD), and immune function [4–6].

The recommended dose of vitamin D for preterm infants differs among advisory bodies. The American Academy of Pediatrics recommends a vitamin D intake of 200–400 IU/day (to convert to μg /day, multiply by 0.025) [3]. In contrast, the European Society for Pediatric Gastroenterology, Hepatology, and Nutrition guidelines suggest a higher intake (800–1000 IU/day) to prevent VDD [2]. These differences may stem from variations in the emphasis on the risks associated with vitamin D deficiency versus excess, differences in the interpretation of results, and regional differences in factors related to vitamin D synthesis. As differing guidelines, there is a growing need for clear and integrated evidence-based recommendations to optimize the health and development of preterm infants in clinical practice. Therefore, it is essential to establish guidelines for vitamin D supplementation that compare high- and low-dose, aiming to identify an optimal regimen that yields better outcomes while minimizing side effects.

Two previous meta-analyses have investigated the effects of varying doses of vitamin D supplementation in preterm infants [7, 8], but their findings remain inconsistent. Yang et al. reported that high-dose supplementation (800–1000 IU/day) did not significantly increase serum 25-hydroxyvitamin D (25[OH]D) levels, compared to the low-dose (400 IU/day) [7]. However, it was associated with improved growth and immune function [7]. In contrast, Kumar et al. found no significant benefits of high-dose supplementation (800–1600 IU/day) on mortality, morbidity, or growth, despite an increase in vitamin D levels [8]. However, these prior studies did not separately evaluate short-term (during hospitalization) and long-term (post-discharge) outcomes, nor did they provide sufficient insight into the dose-response relationship for different clinical endpoints. Given the critical role of vitamin D in skeletal development, growth, and neurodevelopment, it is important to determine whether higher doses confer added benefits−or introduce risks beyond simply correcting deficiency. This is particularly important in preterm infants, who are at increased risk for vitamin D deficiency and its associated complications.

This study aimed to (1) evaluate the short-term and long-term effectiveness and safety of high-dose (≥800 IU/day) and low-dose (<800 IU/day) vitamin D supplementation on preterm infants and (2) provide evidence to help determine the optimal dosage strategy for vitamin D supplementation.

## Methods

This meta-analysis was conducted based on the Preferred Reporting Items for Systematic Reviews and Meta-Analyses (PRISMA guidelines) [9]. The Research protocol was registered and updated in the International Prospective Register of Systematic Reviews. (CRD 42023387565).

### Literature search

The MEDLINE, EMBASE, and Cochrane Library databases were systematically searched for articles published through November 30th, 2024. Medical subject words (MeSH) and free-text terms were used for retrieval. The search terms employed encompassed phrases such as “Infant,” “Premature,” and “Vitamin D.” Supplementary Fig. 1 provides detailed information.

### Study selection

EndNote software was employed for literature management. Qualified studies were identified and cross-checked by two researchers (S.H.S. and H.J.K.). In cases where consensus was needed, a third reviewer (J.S.H.) was consulted.

### Inclusion criteria

The Population, Intervention, Comparison, Outcomes, and Study (PICOS) design criteria was used in searching the literature [10, 11]. The population(P) of interest consisted of preterm infants (gestational age < 37 weeks); The intervention (I) was high-dose vitamin D_3_ (≥800 IU/day) supplementation started during neonatal intensive care unit (NICU) stay after birth; the comparison (C) was low-dose vitamin D_3_ (<800 IU/day) supplementation started during NICU stay after birth; the outcomes (O) include short-term (before 40 weeks’ postmenstrual age [PMA] or at discharge) and long-term (after 40 weeks’ PMA or at the outpatient clinic follow-up) outcomes. Short-term outcomes include serum 25(OH)D level, VDD(serum 25[OH]D level < 20 ng/mL, to convert to nmol/L, multiply by 2.5) [12], vitamin D excess (VDE), skeletal bone mineralization, clinical outcomes (growth, RDS, BPD, late-onset sepsis [LOS], length of hospital stay, and mortality), biochemical markers (parathyroid hormone [PTH], calcium [Ca], phosphorus, alkaline phosphatase [ALP], osteocalcin, and urine calcium/creatinine ratio [uCa/Cr]). Long-term outcomes include serum 25(OH)D level, bone mineral density, mortality, and neurodevelopment; the study design(S) was a randomized controlled trial.

### Exclusion criteria

The studies of usage of different forms of vitamin D (example: vitamin D_2_), selective vitamin D supplementation based on limited criteria (example: vitamin D level <20 ng/mL), or enteral vitamin D supplementation for the treatment of any disease were excluded.

### Data extraction

A predefined data extraction form was used. Ambiguities in data extraction were resolved after discussion with a third reviewer. The following information was extracted: (1) First author and year of publication; (2) country; (3) gestational age at birth; (4) vitamin D dose; (5) sample size; (6) starting point of supplementation; (7) duration or endpoint of supplementation; (8) timing of outcome assessment; (9) primary outcomes: short- and long-term outcomes. Data was extracted by two independent reviewers (S.H.S. and H.J.K.) and reexamined by a third reviewer (H.J.S.).

### Quality assessment

The quality of the included studies was evaluated and cross-checked by two researchers according to the Revised Cochrane risk-of-bias (ROB) tool 2 for randomized trials [13, 14], and a third reviewer was consulted when necessary. A sensitivity analysis was conducted to determine whether the ROB, funding/conflict of interest status, feeding type, publication year, and Mantel-Haenszel analysis as odds ratio influenced the statistical results (Supplementary Table 1). For sensitivity analysis based on feeding type, studies were categorized into two groups: 1) “feeding clearly described and total vitamin D intake estimated” – this group included studies either quantified total vitamin D intake from all sources (supplementation and feeding), or involved exclusively breastfed infants (assuming negligible vitamin D intake from breast milk) or infants fed with formula clearly stated to contain no vitamin D, 2) “feeding type mentioned, but vitamin D intake not estimated” – this group included studies that described the feeding type (e.g., breast milk or formula) and. in some cases, mentioned the use of vitamin D-containing fortifiers or preterm formula, but did not specify the vitamin D content of the feeding source or provide sufficient information to estimate the total vitamin D intake. A funnel plot was used to investigate publication bias.

### Statistical analysis

The outcomes of this review were categorized into short- and long-term outcomes. The definitions of outcome measures in each study are provided in Supplementary Table 2. Statistical analysis was performed using RevMan Web and stata/MP 15.0, and *P* < 0.05 was considered to be statistically significant. The risk difference (RD) and mean difference (MD) were used as outcome estimation measures for categorical and continuous outcomes, respectively. The outcomes were analyzed using a fixed-effects model due to the homogeneity of the preterm infant populations in each study. The results were visually presented using forest plots. The *I*^2^ statistic and Cochran’s Q test were used to assess the statistical heterogeneity. Data that were initially presented as median, interquartile range, and minimum and maximum values were transformed into mean and standard deviation values according to the *Cochrane Handbook* [14]. The certainty of evidence for each outcome was assessed independently by two review authors using the Grading of Recommendations Assessment, Development, and Evaluation (GRADE) System [15]. GRADEPro GDT, a web-based tool, was used to create a “Summary of Findings” table to report the certainty of evidence.

## Results

### Study selection

**Fig. 1:**
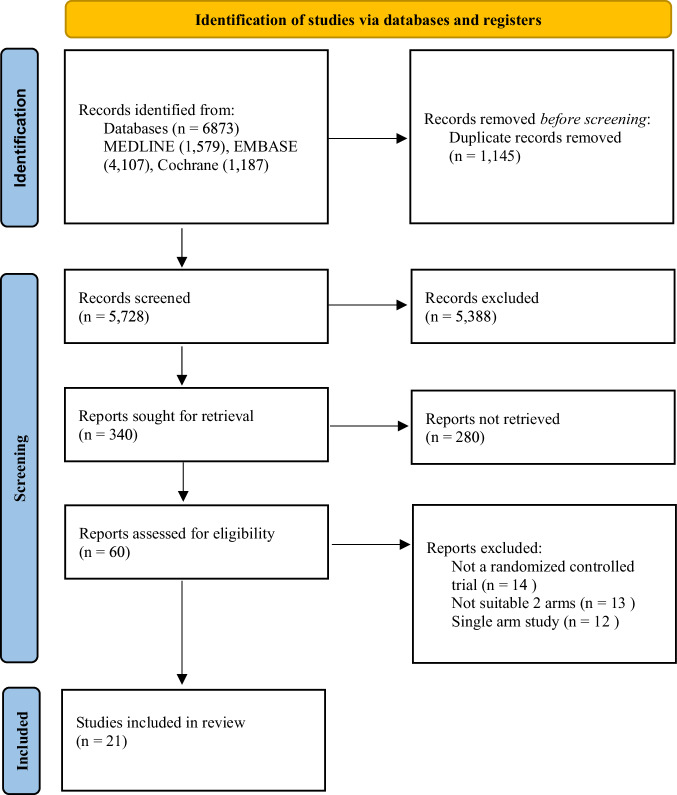
Search strategy flow diagram of literature search and filtering results for a systematic review of the short-term (before 40 weeks’ postmenstrual age or at discharge) and long-term (after 40 weeks’ postmenstrual age or at the outpatient clinic follow-up) effects of vitamin D supplementation for preterm infants, according to the Preferred Reporting Items for Systematic Reviews and Meta-Analyses (PRISMA) 2020 guidelines.

A total of 6873 studies were identified (Fig. 1). Following the removal of duplicates and the subsequent screening of titles, abstracts, and full texts, 21 studies from nine countries (Canada, Egypt, Finland, India, Iran, Israel, Turkey, UK and USA), reporting on 1,130 infants met the final inclusion criteria [16–36]. Three separate studies from one cohort were included, each investigating different outcomes with intervals of several years [20, 25, 35].Additionally, for one study [28], only the abstract was available. The baseline characteristics of studies are summarized in Table 1.

**Table 1:** Study characteristics of 21 randomized-controlled trials analyzing the effects of different doses of vitamin D supplementation for preterm infants.

Study (First author, year)	Country	Gestation (weeks)	Vitamin D dose (IU/day)		Starting point of supplementation	Duration or endpoint of supplementation	Timing of outcome assessment	Primary outcomes		Exclusion criteria
			High-dose (*n*)	Low-dose (*n*)				Short-term	Long-term	
Robinson [34]	United Kingdom	Preterm	1000 (9)	400 (9)	Postnatal 15 days	PMA 39 weeks	Postnatal 14 days, PMA 36 weeks, 39 weeks	25(OH)D	-	Not described
Evans [24]	Canada	Birth weight < 1500 g	2000 (41)	400 (40)	Postnatal 72 hours	6 weeks	2, 4, 6 weeks	Ca, P, ALP, uCa/Cr, 25(OH)D, radiologic score	-	Major congenital anomaly, congenital infection, or inherited metabolic disease. Infants who did not survive to 6 weeks of postnatal age or developed prolonged obstructive jaundice
Pittard [33]	USA	Low birth weight preterm	800 (9)	400 (8)	Within postnatal 12 hours	16 weeks	Biweekly, 16 weeks	25(OH)D	25(OH)D	Minima respiratory disease. Infants who did not reach solely enteral feeding-420J/kg per day or more by 2 weeks of age
Koo [30]	USA	Birth weight ≤ 1500 g	800 (21)	400 (21), 200 (20)	Clinically stable, recovering from pre-existing respiratory illness, not receiving chronic diuretic therapy, tolerating enteral nutrition 75 kcal/kg/day, weight gain at full enteral nutrition	NICU discharge or 2-kg body weight	Termination of formula feeding	Vitamin D and biochemical metabolites	-	Major congenital malformation, necrotizing enterocolitis, major abdominal surgery, chronic diuretic therapy or failure to tolerate feeding for 7 consecutive days after commencement of the study.
Backström, 1999a	Finland	<33	960 (18)	200/kg~400 (21)	Full enteral nutrition	3 months old	6 and 12 weeks, Corrected age of 3 and 6 months	Vitamin D metabolites	Vitamin D metabolites, bone densitometry	Major congenital malformation, failure to supplement vitamin D according to protocol
Backström, 1999b	Finland	<37	1000 (36)	500 (34)	Full enteral nutrition	3 months old	3 months old, 9–11 years	-	Vitamin D metabolites, bone densitometry	Major congenital malformation, failure to supplement vitamin D according to protocol
Alizade [17]	Iran	<38	1000 (36)	400 (32)	Full enteral nutrition	body weight 3000–3500 g	Postnatal 9 weeks	Ca, P, ALP, wrist X-ray	-	Maternal specific medication (anticonvulsants, diuretics, corticosteroids), maternal diabetes mellitus, SGA baby, chronic use of furosemide, NPO for more than 2 weeks, failure of taking vitamin D supplements according to the protocol
Kislal [29]	Turkey	<33	800/kg (11)	400/kg (15), 200/kg (11)	Postnatal 15 days	Postnatal 30 days	15 days after supplementation	Ca, P, ALP, osteocalcin and urinary deoxypyridinoline	-	Congenital malformation and failure to supplement vitamin D according to protocol.
Natarajan [32]	India	28–34	800 (42)	400 (45)	Enteral nutrition ≥100 mL/kg/day by postnatal 2 weeks	Corrected age of 3 months	PMA 40 weeks, Corrected age of 3 months	VDD	VDD	Major malformations, those who received parenteral nutrition for ≥2weeks, or born to mothers receiving phenytoin therapy or with HIV infection
Fort, 2016^a^	USA	23–27	800 (30)	200 (34)	During postnatal 7 days and within 72 hours after initiating enteral nutrition	Postnatal 28 days	Postnatal 28 days	25(OH)D, total number of days alive and off respiratory support	-	Major congenital or chromosomal anomalies, moribund infant with low likelihood of survival as outborn infants, necrotizing enterocolitis Bell’s stage II or greater, spontaneous intestinal perforation, or if feeds were stopped for more than 24 h by the clinical team.
Mathur [31]	India	<37 and Birth weight < 1500 g	1000 (25)	400 (25)	Enteral nutrition ≥100 mL/kg/day	.	6 weeks after supplementation	Ca, P, ALP, 25(OH)D, PTH	-	Major congenital malformations or those not tolerating at least 100 ml/kg/day enteral feeds by day 10 of life.
Hanson [27]	USA	<32	800 (16)	400 (16)	As per unit protocol	As per unit protocol	4 weeks, 8 weeks	Vitamin D metabolites	-	Congenital anomaly, gastrointestinal, liver, or kidney disease, inborn errors of metabolism, parathyroid disease, disorders of calcium metabolism, and infants receiving seizure medication or steroids
Tergestina [36]	India	27–34	1000 (60)	400 (60)	Enteral nutrition ≥100 mL/kg	PMA 40 weeks	PMA 40 weeks	VDD	-	Major congenital anomaly, maternal condition or medications likely to influence vitamin D or calcium metabolism and neonates not attaining 100 ml/kg feeds by 14 days of life
Anderson-Berry [19]	USA	24–32	800 (16)	400 (16)	Initiation of enteral nutrition	NICU discharge	4 weeks and 8 weeks after supplementation	25(OH)D, PTH, Ca, DEXA	-	Congenital anomaly, gastro-intestinal, liver, or kidney disease, inborn errors of metabolism, parathyroid disease, disorders of calcium metabolism, and infants receiving seizure medication or steroid
Bozkurt [23]	Turkey	24–32	1000 (40), 800 (41)	400 (40)	75% of total nutrition by enteral nutrition in potnatal 2 weeks	PMA 36 weeks	PMA 36 weeks	VDD, 25(OH)D	-	Perinatal asphyxia, major congenital or chromosomal anomalies, twin-twin transfusion syndrome, requirement of dopamine >15ug/kg/min or more than inotrope, those with no expectation of survival in first 2 weeks and those that total parenteral nutrition was not ceased by the first 2 weeks
Salas, 2018^a^	USA	23–27	800 (20)	200 (22)	During postnatal 7 days and within 72 hours after initiating enteral nutrition	Postnatal 28 days	22–26 months	-	BSID-III cognitive composite score	Major congenital or chromosomal anomalies, moribund infant with low likelihood of survival as outborn infants, necrotizing enterocolitis Bell’s stage II or greater, spontaneous intestinal perforation, or if feeds were stopped for more than 24 h by the clinical team
Abdel-Hady [16]	Egypt	28–36	800 (25)	400 (25)	Postnatal >72 hours	NICU discharge	1 week after supplementation, PMA 40 weeks	TNF-a, Interleukin-6	-	Major congenital anomalies, chromosomal anomalies, known inborn errors of metabolism, and immunodeficiency disorders
Aly [18]	Egypt	28–33	800 (20)	400 (20)	Enteral nutrition ≥100 mL/kg/day	4 weeks	1 week and 4 weeks after supplementation	T regulatory cells	-	Congenital and chromosomal anomalies, necrotizing enterocolitis, infants who were not fed for >24 hours
Kishore, 2019^b^	India	28–36	800 (46)	400 (46)	Unknown	Unknown	PMA 40 weeks	VDD, vitamin D level	-	Not described
Golan-Tripto [26]	Israel	32–36	800 (25)	400 (25)	Within postnatal 72 hours	12 months	6 months, 12 months	-	25(OH)D, respiratory morbidity	Not described
Aristizabal, 2023^a^	USA	≤28	800 (23)	200 (19)	During postnatal 7 days and within 72 hours after initiating enteral nutrition	Postnatal 28 days	Postnatal 28 days, PMA 36 wks	25(OH)D, Ca, Predictive risk of BPD, postnatal growth faltering, stunting	-	Major congenital or chromosomal anomalies, moribund infant with low likelihood of survival as outborn infants, necrotizing enterocolitis Bell’s stage II or greater, spontaneous intestinal perforation, or if feeds were stopped for more than 24 h by the clinical team.

*ALP* alkaline phosphatase, *BPD* bronchopulmonary dysplasia, *BSID-III* Bayley Scales of Infant and Toddler Development, third edition, Ca calcium, *DEXA* dual-energy X-ray absorptiometry, *NICU* neonatal intensive care unit, *P* phosphorus, *PMA* postmenstrual age, *PTH* parathyroid hormone, *TNF* tumor necrosis factor, uCa/Cr urine calcium/creatinine ratio, *VDD* vitamin D deficiency, *25(OH)D* 25-hydroxyvitamin D.

^a^These three studies were derived from one randomized controlled trial.

^b^Abstract only.

### Short-term outcomes

**Fig. 2:**
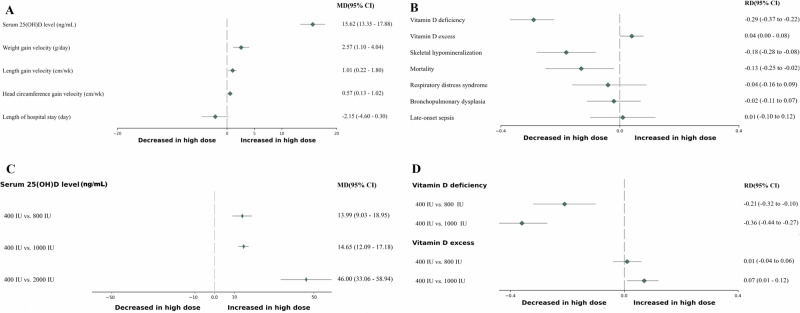
Forest plots comparing the short-term (before 40 weeks’ postmenstrual age or at discharge) outcomes between the high-dose and low-dose vitamin D supplementation for preterm infants. **A** Short-term outcomes: continuous variables. **B** Short-term outcomes: categorical variables. **C** Subgroup analysis of serum 25-hydroxyvitamin D levels according to different doses among the high-dose vitamin D supplementation group. **D** Subgroup analysis of categorical variables according to different doses among the high-dose vitamin D supplementation group. *CI* confidence interval, MD mean difference, *RD* risk difference; *25(OH)D* 25-hydroxyvitamin D.

The Serum 25(OH)D levels and associated outcomes are presented in Fig. 2A, B, Supplementary Fig. 2, and Table 2A. Serum 25(OH)D levels were significantly increased in the high-dose group compared to the low-dose group (MD 15.62; 95% confidence interval [CI] 13.35-17.88; *I*^2^ = 90% [95% CI 88–98]; low certainty of evidence; 13 trials, 739 participants) [16, 19, 21–23, 25, 28, 30–34, 36]. In addition, the risk of VDD was significantly lower in the high-dose group (RD−0.29; 95% CI−0.37 to −0.22; *I*^2^ = 78% [95% CI 48–91]; moderate certainty of evidence; 5 trials, 449 participants) [23, 28, 31, 32, 36]. Moreover, significant difference was not found in the risk of VDE (RD 0.04; 95% CI 0.00–0.08; *I*^2^ = 21% [95% CI 0–88]; low certainty of evidence; 4 trials, 302 participants) [16, 19, 23, 36].

**Table 2:** Summary of findings of short-term (before 40 weeks’ post menstrual age or discharge from neonatal intensive care unit) and long-term (after 40 weeks’ postmenstrual age or from follow up to outpatient clinic after discharge) outcomes between the high-dose and low-dose vitamin D supplementation for preterm infants.

B. Long-term outcomes						
Outcomes	Illustrative comparative risks (95% CI)		Relative effect (95% CI)	No. of Participants (studies)	Quality of the evidence (GRADE)	Comments
	Assumed risk	Corresponding risk				
	Low dose	High dose				
Serum 25(OH)D level (ng/mL)		The mean serum 25(OH)D level in the high-dose groups was 15.62 higher (13.35 to 17.88 higher)	MD 15.62 (13.35 to 17.88)	739 (13 studies)	⊕⊕⊝⊝ low	
Vitamin D deficiency	495 per 1000	205 per 1000 (125 to 275)	RD −0.29 (−0.37 to −0.22)	449 (5 studies)	⊕⊕⊕⊝ moderate	
Vitamin D excess	0 per 1000	40 per 1000 (0 to 80)	RD 0.04 (0.00 to 0.08)	302 (4 studies)	⊕⊕⊝⊝ low	
Skeletal hypomineralization	293 per 1000	113 per 1000 (13 to 213)	RD −0.18 (−0.28 to −0.08)	168 (4 studies)	⊕⊕⊝⊝ low	
Weight gain velocity (g/day)		The mean weight gain velocity in the high-dose groups was 2.57 higher (1.10 to 4.04 higher)	MD 2.57 (1.10 to 4.04)	112 (2 studies)	⊕⊕⊝⊝ low	
Length gain velocity (cm/wk)		The mean length gain velocity in the high-dose groups was 1.01 higher (0.22 to 1.80 higher)	MD 1.01 (0.22 to 1.80)	112 (2 studies)	⊕⊕⊝⊝ low	
Head circumference gain velocity (cm/wk)		The mean head circumference gain velocity in the high-dose groups was 0.57 higher (0.13 lower to 1.02 higher)	MD 0.57 (0.13 to 1.02)	112 (2 studies)	⊕⊕⊝⊝ low	
Respiratory distress syndrome	281 per 1000	241 per 1000 (121 to 371)	RD −0.04 (−0.16 to 0.09)	250 (2 studies)	⊕⊕⊝⊝ low	
Bronchopulmonary dysplasia	281 per 1000	261 per 1000 (171 to 351)	RD −0.02 (−0.11 to 0.07)	316 (5 studies)	⊕⊕⊝⊝ low	
Late-onset sepsis	203 per 1000	213 per 1000 (103 to 323)	RD 0.01 (−0.10 to 0.12)	185 (2 studies)	⊕⊝⊝⊝ very low	
Mortality	186 per 1000	56 per 1000 (0 to 166)	RD −0.13 (−0.25 to −0.02)	114 (2 studies)	⊕⊕⊝⊝ low	
Length of hospital stay (day)		The mean hospital day in the high-dose groups was 2.15 lower (4.60 lower to 0.30 higher)	MD −2.15 (−4.60 to 0.30)	243 (4 studies)	⊕⊕⊕⊝ moderate	
**B. Long-term outcomes**						
**Outcomes**	**Illustrative comparative risks (95% CI)**		**Relative effect (95% CI)**	**No. of participants (studies)**	**Quality of the evidence (GRADE)**	**Comments**
	**Assumed risk**	**Corresponding risk**				
	**Low dose**	**High dose**				
25(OH)D (ng/mL)		The mean 25(OH)D in the high-dose groups was 1.21 higher (4.72 lower to 7.13 higher)	MD 1.21 (−4.72 to 7.13)	142 (4 studies)	⊕⊕⊝⊝ low	
Vitamin D deficiency	350 per 1000	120 per 1000 (0 to 300)	RD −0.23 (−0.40 to -0.05)	80 (1 study)	⊕⊕⊝⊝ low	
Vitamin D excess	25 per 1000	55 per 1000 (0 to 115)	RD 0.03 (−0.04 to 0.09)	80 (1 study)	⊕⊕⊝⊝ low	
Bone mineral density (mg/cm^2^)		The mean bone mineral density in the high-dose groups was 0.33 higher (5.47 lower to 6.12 higher)	MD 0.33 (−5.47 to 6.12)	107 (2 studies)	⊕⊝⊝⊝ very low	
Mortality	294 per 1000	164 per 1000 (0 to 374)	RD −0.13 (−0.33 to 0.08)	64 (1 study)	⊕⊕⊝⊝ low	
Cognitive impairment	364 per 1000	304 per 1000 (14 to 584)	RD −0.06 (−0.35 to 0.22)	42 (1 study)	⊕⊕⊝⊝ low	
Language impairment	571 per 1000	451 per 1000 (141 to 751)	RD −0.12 (−0.43 to 0.18)	41 (1 study)	⊕⊕⊝⊝ low	
Neurodevelopmental impairment	435 per 1000	305 per 1000 (15 to 585)	RD −0.13 (−0.42 to 0.15)	43 (1 study)	⊕⊕⊝⊝ low	

*CI* confidence interval, *MD* mean difference, *RD* risk difference, *25(OH)D25*-hydroxyvitamin D

^a^The basis for the **assumed risk** (e.g., the median control group risk across studies) is provided in the footnotes. The **corresponding risk** (and its 95% confidence interval) is based on the assumed risk in the comparison group and the **relative effect** of the intervention (and its 95% CI).

^b^ GRADEPro GDT, a web-based tool, was used to create a “Summary of Findings” table to report the certainty of evidence.

**High quality:** Further research is very unlikely to change our confidence in the estimated effect. **Moderate quality:** Further research is likely to have an important impact on our confidence in the estimated effect and may change the estimate. **Low quality:** Further research is very likely to have an important impact on our confidence in the estimated effect and is likely to change the estimate. **Very low quality:** We are very uncertain about the estimate.

Skeletal hypomineralization is presented in Fig. 2B, Supplementary Fig. 2, and Table 2A. The RD for skeletal hypomineralization was −0.18, indicating that the high-dose group had a significantly lower risk compared to the low-dose group (95% CI−0.28 to −0.08; *I*^2^ = 94% [95% CI 87–97]; low certainty of evidence; 4 trials; 168 participants) [17, 19, 31, 34].

Clinical outcomes are presented in Fig. 2A, B, Supplementary Fig. 2, and Table 2A. Weight gain velocity (g/day), length gain velocity (cm/week), and head circumference gain velocity (cm/week) all demonstrated a significant increase in the high-dose group compared to the low-dose group (weight: MD 2.57; 95% CI 1.10–4.04; length: MD 1.01; 95% CI 0.22-1.80; head: MD 0.57, 95% CI 0.13–1.02; all three outcomes: *I*^2^ = 0%; low certainty of evidence; 2 trials; 112 participants) [30, 31]. The significant differences in clinical outcomes, including RDS, BPD, LOS, and length of hospital stay were not found. However, the risk of mortality was significantly lower in the high-dose group (RD−0.13; 95% CI−0.25 to −0.02; *I*^2^ = 0%; low certainty of evidence; 2 trials; 114 participants) [16, 25].

Biochemical markers are presented in Supplementary Fig. 2. The PTH level(pg/mL, to convert to pmol/L, multiply by 0.106) was significantly lower in the high-dose group (MD−15.76; 95% CI−21.96 to −9.56; *I*^2^ = 84% [95% CI 61–94]; 4 trials, 302 participants) [19, 23, 31, 36]. The levels of the other biochemical markers, did not differ.

To identify the optimal high-dose, the high-dose group was divided into three subgroups, and a subsequent subgroup analysis was performed (Figs. 2C, D, Supplementary Fig. 2). All three high-dose groups (800 IU [16, 19, 23, 25, 27, 30, 33], 960–1000 IU [22, 23, 31, 34, 36], and 2000 IU [24]) demonstrated a significant elevation in serum 25(OH)D levels compared to the low-dose group. The risk of VDD significantly decreased in both the 800 IU [23, 28, 32] and 1000 IU [23, 31] groups compared to the low-dose group. However, the risk of VDE showed a significant increase exclusively in the 1000 IU subgroup [23, 36] (RD 0.07; 95% CI 0.01–0.12; *I*^2^ = 48%; 2 trials; 179 participants) and not in the 800 IU group [16, 19, 23].

### Long-term outcomes

**Fig. 3:**
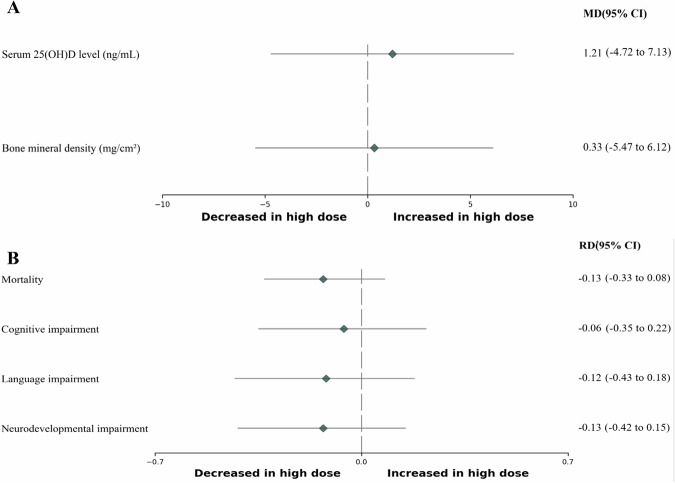
Forest plots comparing the long-term (after 40 weeks’ postmenstrual age or at the outpatient clinic follow-up) outcomes between the high-dose and low-dose vitamin D supplementation for preterm infants. **A** Long-term outcomes: continuous variables. **B** Long-term outcomes: categorical variables. *CI* confidence interval, *MD* mean difference, *RD* risk difference, *25(OH)D* 25-hydroxyvitamin D.

The Serum 25(OH)D levels and associated outcomes are presented in Fig. 3A, Supplementary Fig. 3, and Table 2B. The significant differences in serum 25(OH)D levels were not found between the low-dose and high-dose groups. In a single study focusing on long-term follow-up of VDD and VDE [32], VDD showed a significant decrease in the high-dose group (RD−0.23; 95% CI−0.40 to −0.05; low certainty of evidence; 80 participants), and VDE did not show a significant difference.

Bone mineral density is presented in Fig. 3A, Supplementary Fig. 3, and Table 2B. Bone mineral density (mg/cm^2^) at corrected age 3 months displayed no significant difference (MD 0.33; 95% CI−5.47 to 6.12; *I*^2^ = 62%; very low certainty of evidence; 2 trials; 107 participants) [21, 32]. Bone mineral density at 9–11 years was reported in one study [22], and significant differences were not found between the two groups.

Clinical outcomes are presented in Fig. 3B, Supplementary Fig. 3, and Table 2B. In one study reporting on mortality and neurodevelopmental outcomes at 2 years of age [35], significant differences were not found in terms of mortality, cognitive and language impairment, and total neurodevelopmental impairment.

### Quality assessment and certainty of the evidence

All outcome parameters analyzed are summarized in Table 2. The ROB values are shown in Supplementary Fig. 4. Overall, 25% of the studies had a low risk, 25% raised concerns, and 50% had a high risk. Six studies had concerns regarding bias in the randomization process [17, 20, 24, 29, 33, 34], 9 studies had concerns regarding bias in deviations from the intended interventions [17, 21, 23, 24, 29, 30, 32, 35, 36], and 10 studies had a high risk of bias due to missing outcome data [20, 23–26, 29, 30, 32, 35, 36]. Bias in the measurement of the outcomes was determined to be low across all studies. However, for bias in the selection of the reported results, one was found to have some concerns [25]. To determine the robustness of the ROB, a sensitivity test that excluded the high-risk studies was conducted (Supplementary Table 1). Following the sensitivity analysis for serum 25(OH)D levels, the MD score between the high-dose and low-dose groups decreased (MD 8.11; 95% CI 5.07–11.15). In addition, there was no significant difference in mortality following the sensitivity analysis. Based on the risk of bias, the certainty of the evidence ranges from moderate to very low (Table 2). When analyzing the publication bias concerning different doses of vitamin D supplementation on serum 25(OH)D levels, we visually evaluated the symmetry of the funnel plot shape and found no evidence of asymmetry (Supplementary Fig. 5).

## Discussion

This is the first meta-analysis to simultaneously analyze the short- and long-term outcomes of vitamin D supplementation and suggest an optimal dose for preterm infants. In this study, high-dose vitamin D supplementation showed short-term benefits by increasing serum 25(OH)D levels and growth velocity, and by reducing the risk of VDD, bone hypomineralization, and mortality. Among the high-dose groups, supplementation with 800 IU/day improved serum 25(OH)D levels without increasing the risk of VDE. However, in the long-term, significant differences in serum 25(OH)D levels or clinical outcomes were not found between the high-dose and low-dose groups.

Vitamin D is an essential micronutrient that regulates bone health [37]. Prevention of VDD early in life is important due to its effects on skeletal and non-skeletal health [38, 39]. Our meta-analysis indicates that high-dose vitamin D supplementation is more beneficial for increasing serum 25(OH)D levels and decreasing the risk of VDD and skeletal hypomineralization without VDE before 40 weeks’ PMA or at discharge. Notably, this result is inconsistent with the results of the meta-analysis by Yang et al. [7]. Regarding serum 25(OH)D levels, the meta-analysis included only 5 studies up to 2016 in Yang’s research, whereas our study includes 13 studies up to 2024. Additionally, while the study by Yang et al. did not distinguish between the time points of the outcomes measurements, our study analyzed the short- and long-term outcomes separately. These factors may have contributed to the disparities in our respective results.

Unlike the short-term outcomes, there was insufficient evidence to suggest significant differences in serum 25(OH)D levels and bone mineral density in the long-term. Several possibilities should be considered when interpreting these results. Firstly, for long-term outcomes, only 4 studies were included to evaluate serum 25(OH)D levels. The limited number of studies may have made it challenging to provide an accurate evaluation. Secondly, the timing of the long-term outcomes evaluation varied greatly, ranging from a corrected age of 3 to 12 months. Furthermore, in some studies, the duration of vitamin D supplementation was either inadequately described or was shorter than the latest evaluation time. Thirdly, although medical staff can accurately supplement vitamin D during hospitalization, vitamin D supplementation may be affected by parental compliance after discharge. Fourthly, after discharge, environmental factors such as exposure to sunlight (ultraviolet B-mediated vitamin D formation) [40, 41], the introduction of solid foods containing vitamin D, and the accrual of fat mass (where vitamin D and its metabolites are stored) [42] could affect serum vitamin D levels.

This study showed that high-dose supplementation was more effective in the growth of weight, length, and head circumference over a short-term period. The positive relationship in our study is in line with previous meta-analyses showing that vitamin D supplementation during pregnancy or early infancy was associated with improved infant growth [43, 44]. Notably, vitamin D levels may be related to the levels of insulin-like growth factor 1, which plays a pivotal role in height and weight gain [45, 46]. Moreover, vitamin D plays an important role in the modulation of immune function and oxidative stress, factors that may be linked to growth [6, 47].

In this study, the mortality risk was significantly lower in the high-dose group. The association between vitamin D levels and pulmonary development has been well established [48, 49], and one possible hypothesis is that high-dose vitamin D supplementation is more beneficial for lung maturation and survival. However, following the sensitivity analysis according to the ROB or feeding type, the statistical significance of mortality disappeared. Therefore, the effect of high-dose vitamin D supplementation on mortality may have been overinterpreted. In terms of morbidity (RDS, BPD, late-onset sepsis [LOS], and length of hospital stay), the significant differences were not found in this study. Infants who had not reached enteral feeding were often excluded from study enrollment due to the impracticality of administering vitamin D supplementation. This exclusion may have introduced selection bias, potentially affecting the evaluation of morbidity and mortality outcomes.

When a subgroup analysis of high-dose supplementation stratified by dosage was conducted on the high doses, 800 IU/day was associated with a significant increase in serum 25(OH)D levels and a decrease in VDD during the short-term period. However, an increase in VDE was observed solely in the high-dose group at 1000 IU/day but not at 800 IU/day. To mitigate side effects, it is advisable to keep vitamin D doses as low as possible while still achieving the desired therapeutic effects. Consequently, a vitamin D dose of 800 IU/day could be recommended as the optimal dose to improve short-term outcomes. However, vitamin D levels and complications should be closely monitored simultaneously.

### Limitations

This study had some limitations. Firstly, although 21 studies were included, the number of studies and sample sizes, especially when considering short-term and long-term outcomes, was very small. Accordingly, it is difficult to accurately evaluate the clinical or long-term outcomes in a small number of studies. Secondly, five (25%) of the included studies had ‘some concerns’, and 10 studies (50%) had a high ROB. When a sensitivity analysis was performed by excluding high-risk studies, the MD of serum 25(OH)D decreased from 15.62 to 8.11, though statistical significance was retained (Supplementary Table 1). This suggests that the effect of high-dose vitamin D supplementation on serum 25(OH)D levels may have been overestimated due to selective reporting bias in the high-risk studies. Regarding other outcomes, it is important to acknowledge that sensitivity analysis is limited in its interpretability due to the small number of studies available. Third, this meta-analysis had limited availability and inconsistency of data regarding feeding type and background vitamin D intake. Several included studies did not report detailed information on the vitamin D content from feeding sources such as breast milk, formula, or fortifiers. Although a sensitivity analysis was conducted based on whether total vitamin D intake could be estimated, the number of studies in each subgroup was small, with some subgroups containing only a single study. This limits the ability to assess whether the efficacy of high-dose supplementation differs by feeding context and highlights the need for future trials to report total vitamin D exposure more comprehensively.

## Conclusion

High-dose (≥800 IU/day) vitamin D supplementation for preterm infants was associated with positive short-term outcomes, encompassing improvements in vitamin D levels, growth, and reduced mortality. However, the benefits of high-dose supplementation did not persist in the long-term outcomes. Among the high-dose supplementation, 800 IU/day appeared to be the most appropriate dose. By identifying the optimal and safest dose of vitamin D supplementation for preterm infants, this study could help alleviate the clinical confusion resulting from current guidelines that recommend different doses.

## Supplementary information

Supplementary Fig. 1

Supplementary Fig. 2

Supplementary Fig. 3

Supplementary Fig. 4

Supplementary Fig. 5

Supplementary Table 1

Supplementary Table 2

## Data Availability

The data underlying this meta-analysis are derived from previously published studies, which are all publicly available. No new datasets were generated or analyzed for the current study.
