# Supplementary material for: Short-term and long-term effects of vitamin D supplementation for preterm infants: a systematic review and meta-analysis
Source: J Perinatol. 2025 Oct 7;46(3):425–36. doi: 10.1038/s41372-025-02440-9 (PMC13008753; doi:10.1038/s41372-025-02440-9)
Supplement: Supplementary file 1 — Supplementary Fig. 1 [file 41372_2025_2440_MOESM1_ESM.pdf]

Supplemental Figure 1. Search strategy to find randomized controlled trials comparing of short-term (before 40 weeks' postmenstrual age or at discharge) and long-term (after 40 weeks' postmenstrual age or at the outpatient clinic follow-up) outcomes of different doses of vitamin D supplementation for preterm infants

**MEDLINE search (Date: 2024.11.30)**	
1.	Infant, Premature[MeSH Terms]) OR prematur*[Text Word]) OR preterm[Text Word]) OR pre-matur*[Text Word]) OR pre-term[Text Word] 299,766
2.	Infant, very low birth weight[MeSH Terms]) OR Very Low Birth Weight Infant*[Text Word]) OR "Very Low Birth Weight"[Text Word])) OR "VLBW"[Text Word] 16,869
3.	Infant, Extremely Low Birth Weight[MeSH Terms]) OR Extremely Low Birth Weight Infant*[Text Word]) OR "ELBW"[Text Word] 3,833
4.	1-3/OR 304,615
5.	"Vitamin D"[Mesh] 71,589
6.	"Vitamin D"[TW] OR "VitaminD"[TW] OR "VitD"[TW] OR "25-hydroxyvitamin D"[TW] OR "25-hydroxyergocalciferol"[TW] OR Ergocalciferol*[TW] OR Cholecalciferol*[TW] OR Hydroxycholecalciferol*[TW] OR Calcifediol[TW] OR Dihydroxycholecalciferol*[TW] OR "25(OH)D"[TW] OR "1,25(OH)2-vitD"[TW] 103,194
7.	5-6/OR 110,023
8.	4 AND 7 1,579
**EMBASE search (Date: 2024.11.30)**	
1.	'prematurity'/exp OR prematurity 167,479
2.	prematur*:ab,ti,kw,de 399,419
3.	'pre-mature':ab,ti,kw,de 704
4.	'pre-term':ab,ti,kw,de 5,788
5.	'preterm':ab,ti,kw,de 150,325
6.	'very low birth weight':ab,ti,kw,de 18,716
7.	'very low birth weights':ab,ti,kw,de 161
8.	'extremely low birth weight':ab,ti,kw,de 6,222
9.	'extremely low birth weights':ab,ti,kw,de 53
10.	1-9/OR 435,839
11.	'vitamin D'/exp OR 'vitamin D' 223,337
12.	'vitamin d':ab,ti,kw,de 179,609
13.	'vitamind':ab,ti,kw,de 2,333
14.	'vitd':ab,ti,kw,de 2,681
15.	'25-hydroxyvitamin d':ab,ti,kw,de 37,709
16.	'25-hydroxyergocalciferol':ab,ti,kw,de 1,429
17.	'ergocalciferol*':ab,ti,kw,de 11,109
18.	'cholecalciferol*':ab,ti,kw,de 6,087
19.	'hydroxycholecalciferol*':ab,ti,kw,de 1,936
20.	'calcifediol':ab,ti,kw,de 12,041
21.	'dihydroxycholecalciferol*':ab,ti,kw,de 1,879
22.	'25(oh)d':ab,ti,kw,de 21,720
23.	'1,25(oh)2-vitd':ab,ti,kw,de 58
24.	11-23/OR 224,966

25.	10 AND 24 4,107
**Cochrane search (Date: 2024.11.30)**	
1.	MeSH descriptor: [Infant, Premature] explode all trees 5,721
2.	prematur*:ti,ab,kw (Word variations have been searched) 30,567
3.	pre-matur*:ti,ab,kw (Word variations have been searched) 46
4.	pre-term:ti,ab,kw (Word variations have been searched) 414
5.	preterm:ti,ab,kw (Word variations have been searched) 18,229
6.	MeSH descriptor: [Infant, Very Low Birth Weight] explode all trees 1,372
7.	Very Low Birth Weight:ti,ab,kw (Word variations have been searched) 3,414
8.	VLBW:ti,ab,kw (Word variations have been searched) 1,056
9.	MeSH descriptor: [Infant, Extremely Low Birth Weight] explode all trees 231
10.	Extremely Low Birth Weight:ti,ab,kw (Word variations have been searched) 942
11.	1-10/OR 38,056
12.	MeSH descriptor: [Vitamin D] explode all trees 8,570
13.	Vitamin D:ti,ab,kw (Word variations have been searched) 18,559
14.	VitaminD:ti,ab,kw (Word variations have been searched) 265
15.	VitD:ti,ab,kw (Word variations have been searched) 377
16.	25 hydroxyvitamin D:ti,ab,kw (Word variations have been searched) 3,534
17.	25 hydroxyergocalciferol:ti,ab,kw (Word variations have been searched) 36
18.	ergocalciferol*:ti,ab,kw (Word variations have been searched) 1,821
19.	cholecalciferol*:ti,ab,kw (Word variations have been searched) 3,790
20.	hydroxycholecalciferol*:ti,ab,kw (Word variations have been searched) 451
21.	calcifediol:ti,ab,kw (Word variations have been searched) 644
22.	dihydroxycholecalciferol*:ti,ab,kw (Word variations have been searched) 149
23.	25(OH)D:ti,ab,kw (Word variations have been searched) 41,332
24.	1,25(OH)2 vitD:ti,ab,kw (Word variations have been searched) 17
25.	12-24/OR 55,949
26.	11 AND 25 1,187
