# Supplementary material for: Short-term and long-term effects of vitamin D supplementation for preterm infants: a systematic review and meta-analysis
Source: J Perinatol. 2025 Oct 7;46(3):425–36. doi: 10.1038/s41372-025-02440-9 (PMC13008753; doi:10.1038/s41372-025-02440-9)
Supplement: Supplementary file 2 — Supplementary Fig. 2 [file 41372_2025_2440_MOESM2_ESM.pdf]

Supplemental Figure 2. Forest plot for each short-term (before 40 weeks' post menstrual age or discharge from neonatal intensive care unit) outcome variable comparing high dose(≥800IU/day) and low dose (<800IU/day) vitamin D supplementation for preterm infants. Each study identified by first author and year.

(A) Serum 25-hydroxyvitamin D level (ng/mL)

Study or Subgroup	High dose Mean	High dose SD	High dose Total	Low dose Mean	Low dose SD	Low dose Total	Weight	Mean Difference IV, Fixed, 95% CI
Abdel-Hady 2019	67.4	37.1	25	54.8	35.1	25	1.3%	12.60 [-7.42, 32.62]
Anderson-Berry 2017	55.6	30.65	16	45.71	43.17	16	0.8%	9.89 [-16.05, 35.83]
Backström 1999a	26.72	12.14	11	18.31	7.37	15	7.8%	8.41 [0.32, 16.50]
Bozkurt 2017	41.48	20.13	81	29.4	13	40	14.5%	12.08 [6.13, 18.03]
Evans 1989	73.75	39.75	41	27.75	14.25	40	3.1%	46.00 [33.06, 58.94]
Fort 2016	78.5	34.8	30	40.7	23	34	2.4%	37.80 [23.14, 52.46]
Hanson 2016	42.1	15.36	16	32.5	11.7	16	5.7%	9.60 [0.14, 19.06]
Koo 1995	31.25	35.74	21	29.12	65.4	41	0.8%	2.13 [-23.06, 27.32]
Mathur 2016	50.9	11.1	25	29.7	11	25	13.7%	21.20 [15.07, 27.33]
Natarajan 2014	42.4	68.3	42	20.9	29	45	1.0%	21.50 [-0.83, 43.83]
Pittard Iii 1991	48	31	9	24	5	8	1.2%	24.00 [3.45, 44.55]
Robinson 1981	15.06	3.24	9	15.58	6.12	9	25.0%	-0.52 [-5.04, 4.00]
Tergestina 2016	47.47	14.42	51	17.48	9.27	48	22.7%	29.99 [25.24, 34.74]
**Total (95% CI)**			**377**			**362**	**100.0%**	**15.62 [13.35, 17.88]**

Heterogeneity: Chi² = 125.48, df = 12 (P < 0.00001); I² = 90%
Test for overall effect: Z = 13.52 (P < 0.00001)

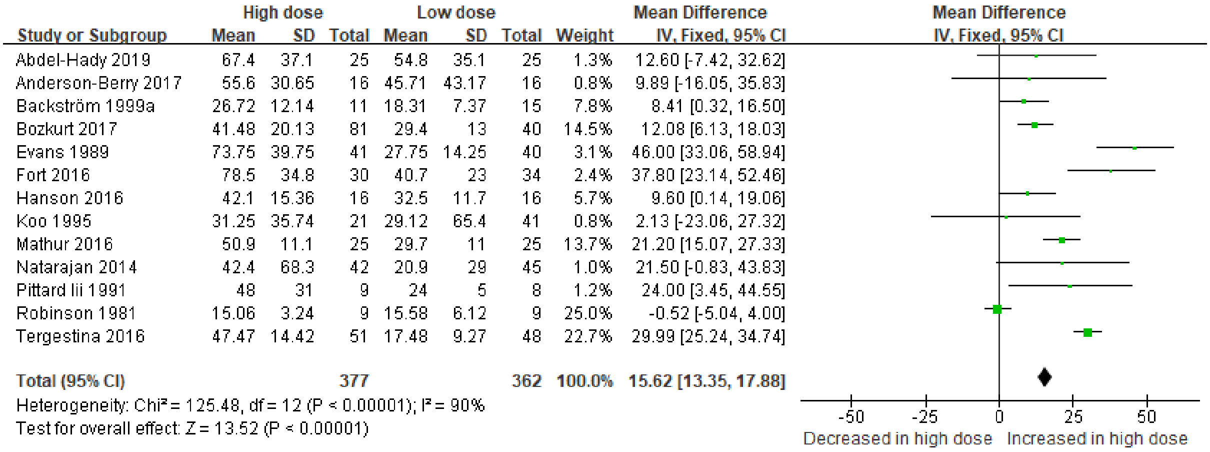

(B) Vitamin D deficiency

Study or Subgroup	High dose Events	High dose Total	Low dose Events	Low dose Total	Weight	Risk Difference M-H, Fixed, 95% CI
Bozkurt 2017	5	81	9	40	24.6%	-0.16 [-0.30, -0.02]
Kishore 2019	19	46	29	46	21.2%	-0.22 [-0.42, -0.02]
Mathur 2016	0	25	5	25	11.5%	-0.20 [-0.37, -0.03]
Natarajan 2014	16	42	30	45	20.0%	-0.29 [-0.49, -0.08]
Tergestina 2016	1	51	28	48	22.7%	-0.56 [-0.71, -0.42]
**Total (95% CI)**		**245**		**204**	**100.0%**	**-0.29 [-0.37, -0.22]**
Total events	41		101			

Heterogeneity: Chi² = 18.55, df = 4 (P = 0.0010); I² = 78%
Test for overall effect: Z = 7.42 (P < 0.00001)

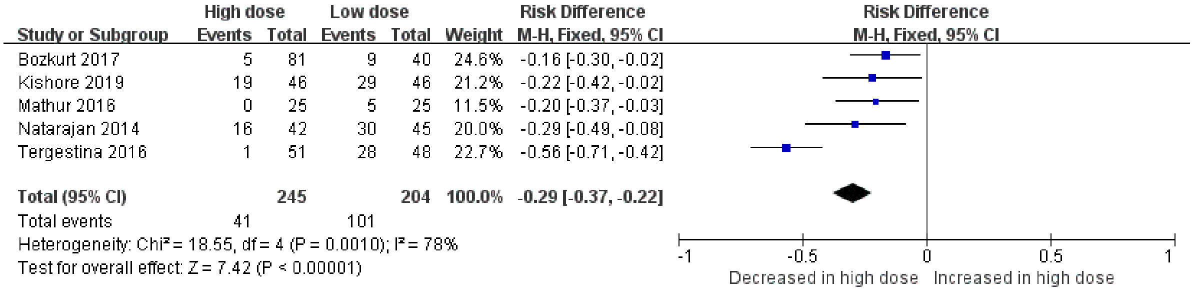

(C) Vitamin D excess

Study or Subgroup	High dose Events	High dose Total	Low dose Events	Low dose Total	Weight	Risk Difference M-H, Fixed, 95% CI
Abdel-Hady 2019	0	25	0	25	17.4%	0.00 [-0.07, 0.07]
Anderson-Berry 2017	0	16	0	16	11.1%	0.00 [-0.11, 0.11]
Bozkurt 2017	2	81	0	40	37.2%	0.02 [-0.03, 0.07]
Tergestina 2016	5	51	0	48	34.3%	0.10 [0.01, 0.19]
**Total (95% CI)**		**173**		**129**	**100.0%**	**0.04 [0.00, 0.08]**
Total events	7		0			

Heterogeneity: Chi² = 3.82, df = 3 (P = 0.28); I² = 21%
Test for overall effect: Z = 2.05 (P = 0.04)

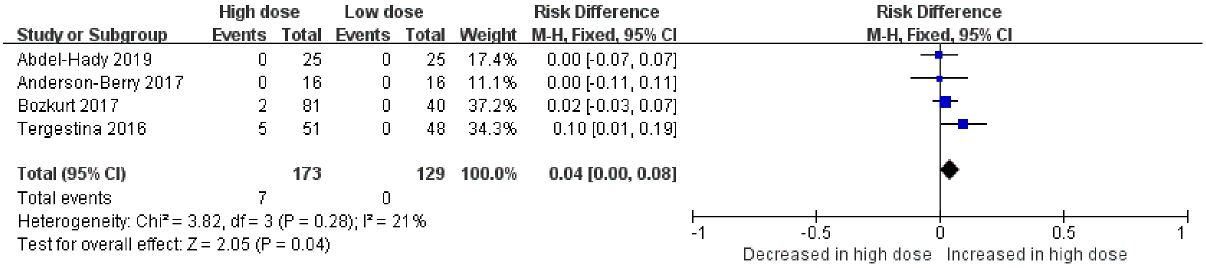

(D) Skeletal hypomineralization

Study or Subgroup	High dose Events	High dose Total	Low dose Events	Low dose Total	Weight	Risk Difference M-H, Fixed, 95% CI
Alizade 2006	0	36	0	32	40.4%	0.00 [-0.06, 0.06]
Anderson-Berry 2017	3	16	9	16	19.1%	-0.38 [-0.68, -0.07]
Mathur 2016	6	25	15	25	29.8%	-0.36 [-0.61, -0.11]
Robinson 1981	0	9	0	9	10.7%	0.00 [-0.19, 0.19]
**Total (95% CI)**		**86**		**82**	**100.0%**	**-0.18 [-0.28, -0.08]**
Total events	9		24			

Heterogeneity: Chi² = 46.21, df = 3 (P < 0.00001); I² = 94%
Test for overall effect: Z = 3.45 (P = 0.0006)

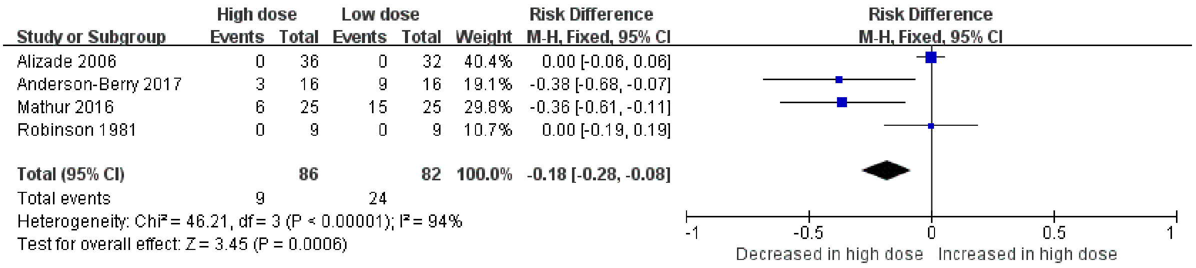

(E) growth

Study or Subgroup	High dose Mean	High dose SD	High dose Total	Low dose Mean	Low dose SD	Low dose Total	Weight	Mean Difference IV, Fixed, 95% CI
**Weight gain velocity (g/day)**								
Koo 1995	31.2	5.04	21	29.09	4.52	41	32.9%	2.11 [-0.45, 4.67]
Mathur 2016	16.4	3.7	25	13.6	2.7	25	67.1%	2.80 [1.00, 4.60]
**Subtotal (95% CI)**			**46**			**66**	**100.0%**	**2.57 [1.10, 4.04]**
Heterogeneity: Chi² = 0.19, df = 1 (P = 0.67); I² = 0%								
Test for overall effect: Z = 3.43 (P = 0.0006)								
**Length gain velocity (cm/week)**								
Koo 1995	11.9	2.6	21	11.2	2.1	41	37.9%	0.70 [-0.58, 1.98]
Mathur 2016	7.9	2	25	6.7	1.6	25	62.1%	1.20 [0.20, 2.20]
**Subtotal (95% CI)**			**46**			**66**	**100.0%**	**1.01 [0.22, 1.80]**
Heterogeneity: Chi² = 0.36, df = 1 (P = 0.55); I² = 0%								
Test for overall effect: Z = 2.50 (P = 0.01)								
**Head circumference gain velocity (cm/week)**								
Koo 1995	11.9	1.4	21	11.2	1.4	41	37.1%	0.70 [-0.04, 1.44]
Mathur 2016	6.8	0.8	25	6.3	1.2	25	62.9%	0.50 [-0.07, 1.07]
**Subtotal (95% CI)**			**46**			**66**	**100.0%**	**0.57 [0.13, 1.02]**
Heterogeneity: Chi² = 0.18, df = 1 (P = 0.67); I² = 0%								
Test for overall effect: Z = 2.51 (P = 0.01)								

Test for subgroup differences: Chi² = 6.83, df = 2 (P = 0.03), I² = 70.7%

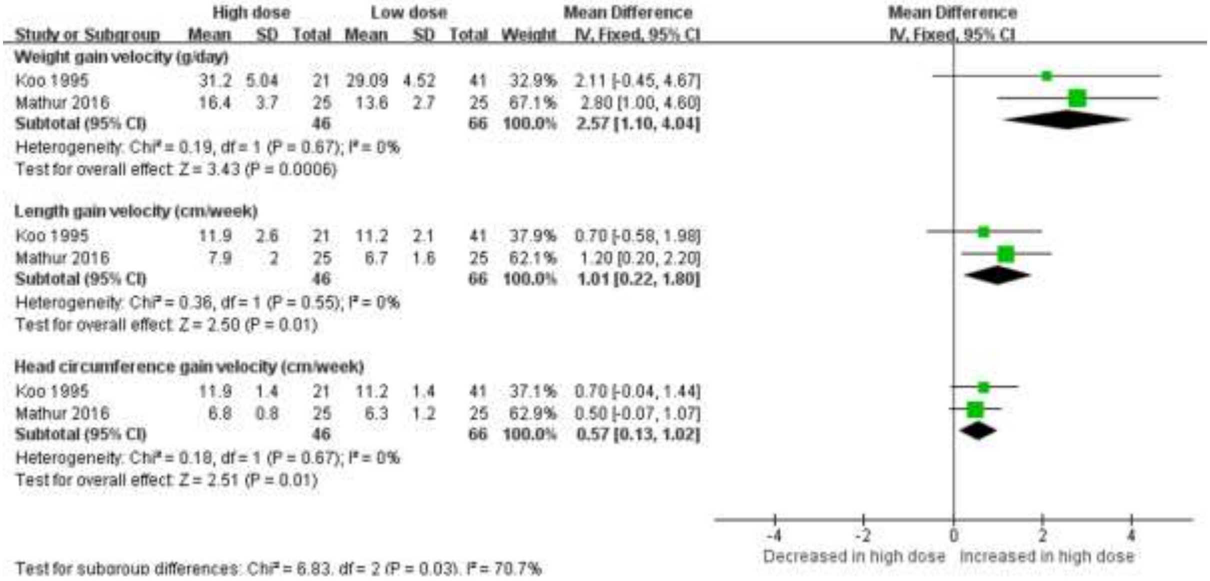

(F) Respiratory distress syndrome (RDS), bronchopulmonary dysplasia (BPD), and late-onset sepsis (LOS)

Study or Subgroup	High dose Events	High dose Total	Low dose Events	Low dose Total	Weight	Risk Difference M-H, Fixed, 95% CI
**RDS**						
Bozkurt 2017	44	81	23	40	62.7%	-0.03 [-0.22, 0.16]
Fort 2016	27	30	32	34	37.3%	-0.04 [-0.17, 0.09]
**Subtotal (95% CI)**		**111**		**74**	**100.0%**	**-0.04 [-0.16, 0.09]**
Total events	71		55			
Heterogeneity: Chi² = 0.01, df = 1 (P = 0.93); I² = 0%						
Test for overall effect: Z = 0.54 (P = 0.59)						
**BPD**						
Abdel-Hady 2019	0	25	0	25	16.6%	0.00 [-0.07, 0.07]
Aristizabal 2022	6	23	4	19	13.8%	0.05 [-0.21, 0.31]
Backström 1999a	3	18	4	21	12.9%	-0.02 [-0.26, 0.22]
Bozkurt 2017	17	81	10	40	35.6%	-0.04 [-0.20, 0.12]
Fort 2016	17	30	21	34	21.2%	-0.05 [-0.29, 0.19]
**Subtotal (95% CI)**		**177**		**139**	**100.0%**	**-0.02 [-0.11, 0.07]**
Total events	43		39			
Heterogeneity: Chi² = 0.72, df = 4 (P = 0.95); I² = 0%						
Test for overall effect: Z = 0.46 (P = 0.65)						
**LOS**						
Bozkurt 2017	17	81	4	40	62.7%	0.11 [-0.02, 0.24]
Fort 2016	5	30	11	34	37.3%	-0.16 [-0.36, 0.05]
**Subtotal (95% CI)**		**111**		**74**	**100.0%**	**0.01 [-0.10, 0.12]**
Total events	22		15			
Heterogeneity: Chi² = 4.83, df = 1 (P = 0.03); I² = 79%						
Test for overall effect: Z = 0.18 (P = 0.86)						

Test for subgroup differences: Chi² = 0.31, df = 2 (P = 0.85), I² = 0%

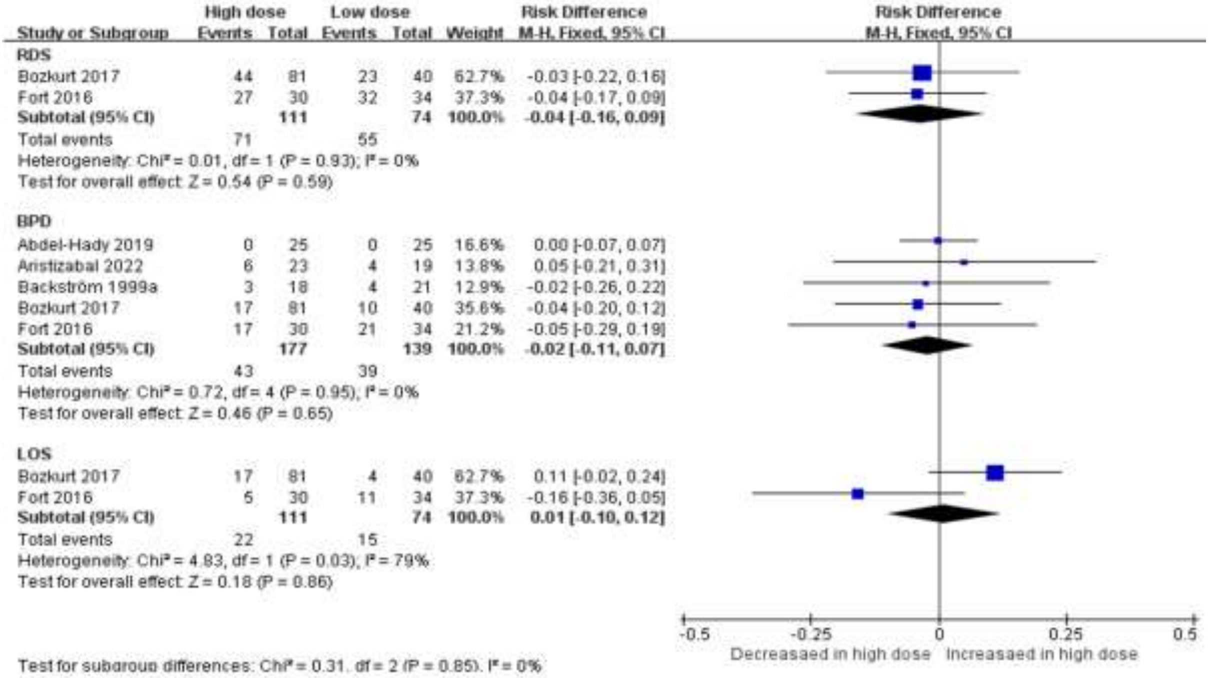

(G) Length of hospital stay (days)

Study or Subgroup	High dose Mean	High dose SD	High dose Total	Low dose Mean	Low dose SD	Low dose Total	Weight	Mean Difference IV, Fixed, 95% CI
Abdel-Hady 2019	32.3	14.7	25	34.3	17.2	25	7.6%	-2.00 [-10.87, 6.87]
Aly 2019	22	3.49	20	24.9	5.14	20	81.1%	-2.90 [-5.62, -0.18]
Anderson-Berry 2017	59	43.33	16	52	15.56	16	1.2%	7.00 [-15.56, 29.56]
Bozkurt 2017	56.7	25.45	81	54	17.4	40	10.1%	2.70 [-5.03, 10.43]
**Total (95% CI)**			**142**			**101**	**100.0%**	**-2.15 [-4.60, 0.30]**

Heterogeneity: Chi² = 2.44, df = 3 (P = 0.49); I² = 0%
Test for overall effect: Z = 1.72 (P = 0.09)

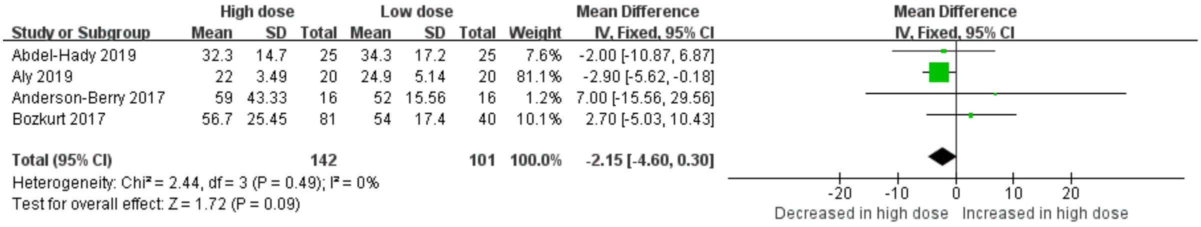

(H) Mortality

Study or Subgroup	High dose Events	High dose Total	Low dose Events	Low dose Total	Weight	Risk Difference M-H, Fixed, 95% CI
Abdel-Hady 2019	2	25	4	25	44.0%	-0.08 [-0.26, 0.10]
Fort 2016	1	30	7	34	56.0%	-0.17 [-0.32, -0.02]
**Total (95% CI)**		**55**		**59**	**100.0%**	**-0.13 [-0.25, -0.02]**
Total events	3		11			

Heterogeneity: Chi² = 0.60, df = 1 (P = 0.44); I² = 0%
Test for overall effect: Z = 2.24 (P = 0.02)

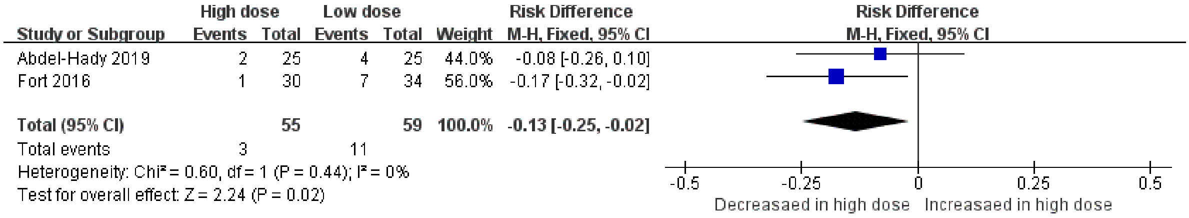

(I) Biochemical markers

Study or Subgroup	High dose Mean	High dose SD	High dose Total	Low dose Mean	Low dose SD	Low dose Total	Weight	Mean Difference IV, Fixed, 95% CI
**Serum PTH**								
Anderson-Berry 2017	40	17.04	16	32.5	21.11	16	21.7%	7.50 [-5.79, 20.79]
Bozkurt 2017	55.04	52.73	81	64.5	57	40	8.6%	-9.46 [-30.53, 11.61]
Mathur 2016	38.6	8.5	25	61	17.5	25	66.0%	-22.40 [-30.03, -14.77]
Tergestina 2016	32.32	50.42	51	81.73	104.32	48	3.6%	-49.41 [-82.00, -16.82]
**Subtotal (95% CI)**			**173**			**129**	**100.0%**	**-15.76 [-21.96, -9.56]**
Heterogeneity: Chi² = 19.11, df = 3 (P = 0.0003); I² = 84%								
Test for overall effect: Z = 4.99 (P < 0.00001)								
**Serum Calcium**								
Abdel-Hady 2019	9.3	1	23	8.5	0.8	21	2.9%	0.80 [0.27, 1.33]
Alizade 2006	9.76	0.45	36	9.865	0.4	32	20.4%	-0.11 [-0.31, 0.10]
Anderson-Berry 2017	10.15	0.37	16	10.3	0.22	16	18.7%	-0.15 [-0.36, 0.06]
Bozkurt 2017	9.45	0.45	81	9.5	0.6	40	18.8%	-0.05 [-0.26, 0.16]
Fort 2016	9.9	0.8	30	10	1.2	34	3.4%	-0.10 [-0.59, 0.39]
Kislal 2008	9.5	1	11	9.64	0.93	26	1.7%	-0.14 [-0.83, 0.55]
Koo 1995	9.62	0.92	21	9.62	1.28	41	2.7%	0.00 [-0.56, 0.56]
Mathur 2016	10.3	0.3	25	9.5	0.5	25	15.9%	0.80 [0.57, 1.03]
Tergestina 2016	9.63	0.47	51	9.42	0.68	48	15.5%	0.21 [-0.02, 0.44]
**Subtotal (95% CI)**			**294**			**283**	**100.0%**	**0.12 [0.03, 0.21]**
Heterogeneity: Chi² = 55.89, df = 8 (P < 0.00001); I² = 86%								
Test for overall effect: Z = 2.55 (P = 0.01)								
**Serum Phosphorus**								
Abdel-Hady 2019	5.5	0.8	23	5.1	0.6	21	16.9%	0.40 [-0.02, 0.82]
Alizade 2006	5.37	0.5	36	5.6	1.2	32	14.7%	-0.23 [-0.68, 0.22]
Bozkurt 2017	5.45	1.02	81	5.6	0.9	40	23.0%	-0.15 [-0.51, 0.21]
Fort 2016	4.5	1.3	30	4.9	1.8	34	5.0%	-0.40 [-1.16, 0.36]
Kislal 2008	3.5	2	11	4.8	1.79	26	1.6%	-1.30 [-2.67, 0.07]
Koo 1995	5.88	0.87	21	6.35	1.6	41	7.7%	-0.47 [-1.09, 0.15]
Mathur 2016	5.7	0.7	25	5.3	0.9	25	14.6%	0.40 [-0.05, 0.85]
Tergestina 2016	5.46	1.08	51	5.73	1.06	48	16.5%	-0.27 [-0.69, 0.15]
**Subtotal (95% CI)**			**278**			**267**	**100.0%**	**-0.06 [-0.23, 0.11]**
Heterogeneity: Chi² = 16.15, df = 7 (P = 0.02); I² = 57%								
Test for overall effect: Z = 0.72 (P = 0.47)								
**Serum alkaline phosphatase**								
Abdel-Hady 2019	847	76.3	23	849.67	68.89	21	24.3%	-2.67 [-45.57, 40.23]
Alizade 2006	608.65	84.5	36	593.75	82.5	32	28.3%	14.90 [-24.84, 54.64]
Bozkurt 2017	532	237.82	81	494	167	40	8.3%	38.00 [-35.22, 111.22]
Kislal 2008	1,254	356.2	11	956.01	338.45	26	0.7%	297.99 [50.54, 545.44]
Koo 1995	232	64.15	21	221.24	149.76	41	15.7%	10.76 [-42.66, 64.18]
Mathur 2016	180.7	67.9	25	259.4	117.6	25	15.8%	-78.70 [-131.93, -25.47]
Tergestina 2016	398.31	182.32	51	415.96	224.89	48	6.8%	-17.65 [-98.59, 63.29]
**Subtotal (95% CI)**			**248**			**233**	**100.0%**	**-3.02 [-24.17, 18.13]**
Heterogeneity: Chi² = 15.82, df = 6 (P = 0.01); I² = 62%								
Test for overall effect: Z = 0.28 (P = 0.78)								
**Urine Ca/Cr ratio**								
Abdel-Hady 2019	0.3	0.2	25	0.3	0.1	25	100.0%	0.00 [-0.09, 0.09]
Bozkurt 2017	0.65	0.55	81	0.7	0.6	40		Not estimable
Koo 1995	0.74	0.55	21	0.68	1.15	41		Not estimable
**Subtotal (95% CI)**			**25**			**25**	**100.0%**	**0.00 [-0.09, 0.09]**
Heterogeneity: Not applicable								
Test for overall effect: Z = 0.00 (P = 1.00)								

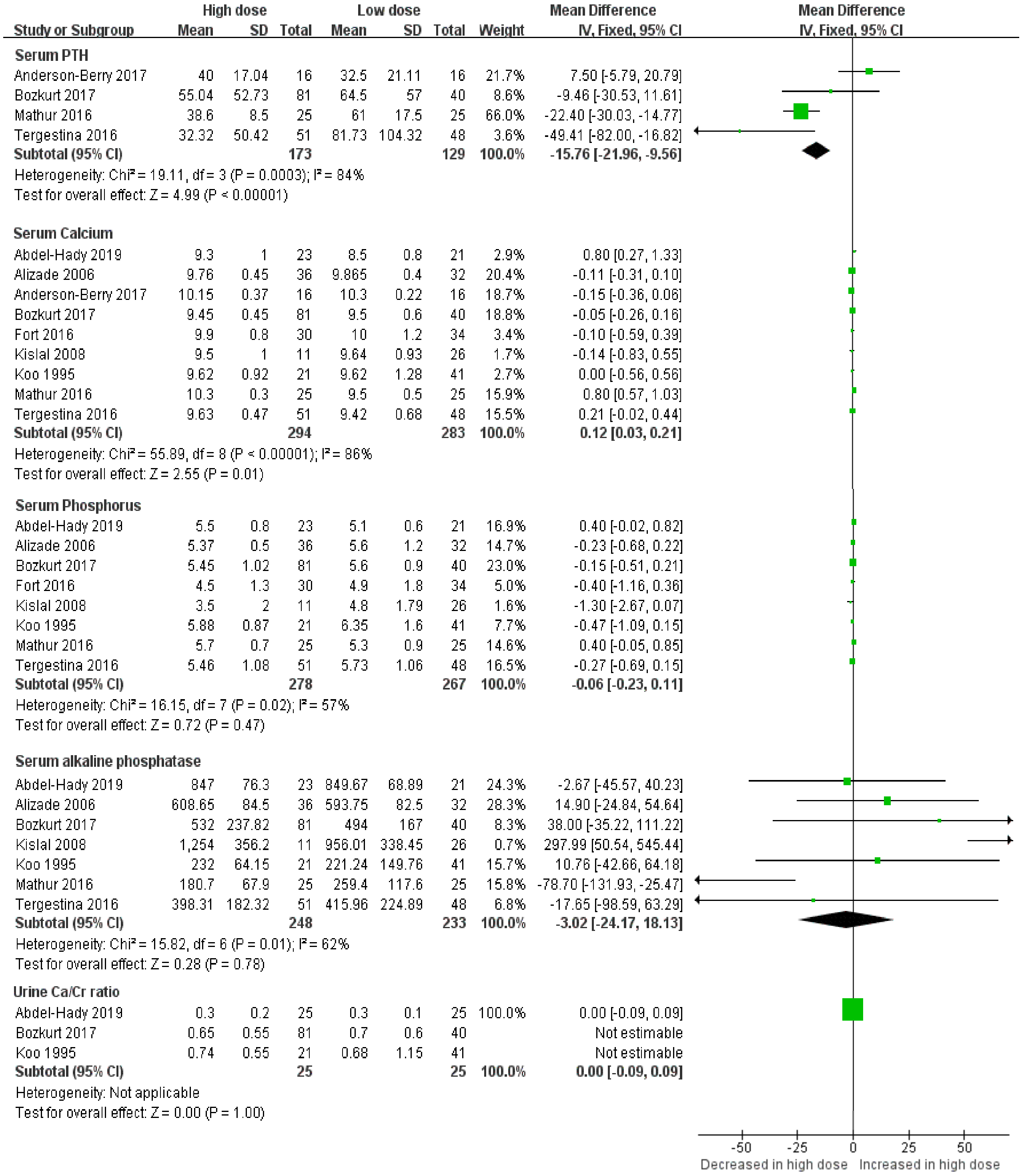

(J) Serum 25-hydroxyvitamin D level according to the different high doses (ng/mL)

Study or Subgroup	Experimental Mean	Experimental SD	Experimental Total	Control Mean	Control SD	Control Total	Weight	Mean Difference IV, Fixed, 95% CI
**800 IU vs 400 IU**								
Abdel-Hady 2019	67.4	37.1	25	54.8	35.1	25	6.1%	12.60 [-7.42, 32.62]
Anderson-Berry 2017	55.6	30.65	16	45.71	43.17	16	3.7%	9.89 [-16.05, 35.83]
Bozkurt 2017	40	21.4	41	29.4	13	40	41.6%	10.60 [2.91, 18.29]
Fort 2016	78.5	34.8	30	40.7	23	34	11.4%	37.80 [23.14, 52.46]
Hanson 2016	42.1	15.36	16	32.5	11.7	16	27.5%	9.60 [0.14, 19.06]
Koo 1995	31.25	35.74	21	29.12	65.4	41	3.9%	2.13 [-23.06, 27.32]
Pittard III 1991	48	31	9	24	5	8	5.8%	24.00 [3.45, 44.55]
**Subtotal (95% CI)**			**158**			**180**	**100.0%**	**13.99 [9.03, 18.95]**
Heterogeneity: Chi² = 13.59, df = 6 (P = 0.03); I² = 56%								
Test for overall effect: Z = 5.53 (P < 0.00001)								
**1000 IU vs 400 IU**								
Backström 1999a	26.72	12.14	11	18.31	7.37	15	9.9%	8.41 [0.32, 16.50]
Bozkurt 2017	43	18.9	40	29.4	13	40	12.8%	13.60 [6.49, 20.71]
Mathur 2016	50.9	11.1	25	29.7	11	25	17.2%	21.20 [15.07, 27.33]
Robinson 1981	15.06	3.24	9	15.58	6.12	9	31.5%	-0.52 [-5.04, 4.00]
Tergestina 2016	47.47	14.42	51	17.48	9.27	48	28.6%	29.99 [25.24, 34.74]
**Subtotal (95% CI)**			**136**			**137**	**100.0%**	**14.64 [12.09, 17.18]**
Heterogeneity: Chi² = 90.06, df = 4 (P < 0.00001); I² = 96%								
Test for overall effect: Z = 11.29 (P < 0.00001)								
**2000 IU vs 400 IU**								
Evans 1989	73.75	39.75	41	27.75	14.25	40	100.0%	46.00 [33.06, 58.94]
**Subtotal (95% CI)**			**41**			**40**	**100.0%**	**46.00 [33.06, 58.94]**
Heterogeneity: Not applicable								
Test for overall effect: Z = 6.97 (P < 0.00001)								

Test for subgroup differences: Chi² = 22.13, df = 2 (P < 0.0001), I² = 91.0%

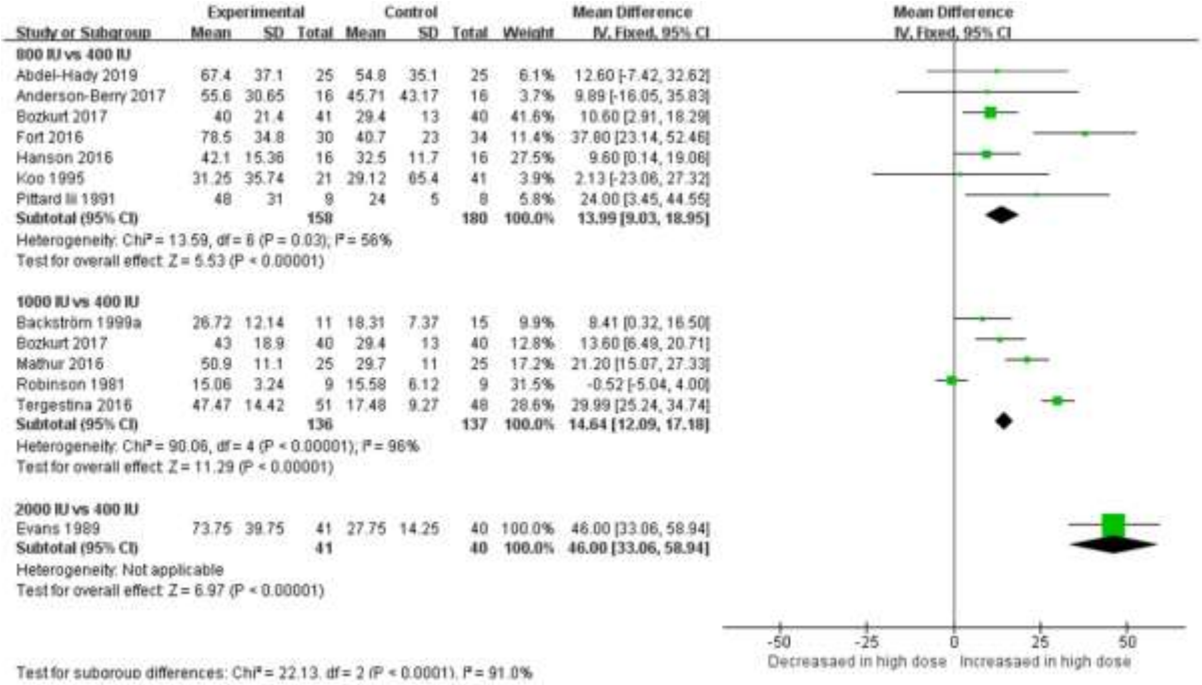

(K) Vitamin D deficiency according to the different high doses

Study or Subgroup	High dose Events	High dose Total	Low dose Events	Low dose Total	Weight	Risk Difference M-H, Fixed, 95% CI
**800 IU vs 400 IU**						
Bozkurt 2017	4	41	9	40	31.2%	-0.13 [-0.29, 0.03]
Kishore 2019	19	46	29	46	35.4%	-0.22 [-0.42, -0.02]
Natarajan 2014	16	42	30	45	33.4%	-0.29 [-0.49, -0.08]
**Subtotal (95% CI)**		**129**		**131**	**100.0%**	**-0.21 [-0.32, -0.10]**
Total events	39		68			
Heterogeneity: Chi² = 1.62, df = 2 (P = 0.45); I² = 0%						
Test for overall effect: Z = 3.81 (P = 0.0001)						
**1000 IU vs 400 IU**						
Bozkurt 2017	1	40	9	40	34.9%	-0.20 [-0.34, -0.06]
Mathur 2016	0	25	5	25	21.8%	-0.20 [-0.37, -0.03]
Tergestina 2016	1	51	28	48	43.2%	-0.56 [-0.71, -0.42]
**Subtotal (95% CI)**		**116**		**113**	**100.0%**	**-0.36 [-0.44, -0.27]**
Total events	2		42			
Heterogeneity: Chi² = 16.27, df = 2 (P = 0.0003); I² = 88%						
Test for overall effect: Z = 8.00 (P < 0.00001)						

Test for subgroup differences: Chi² = 4.12, df = 1 (P = 0.04), I² = 75.7%

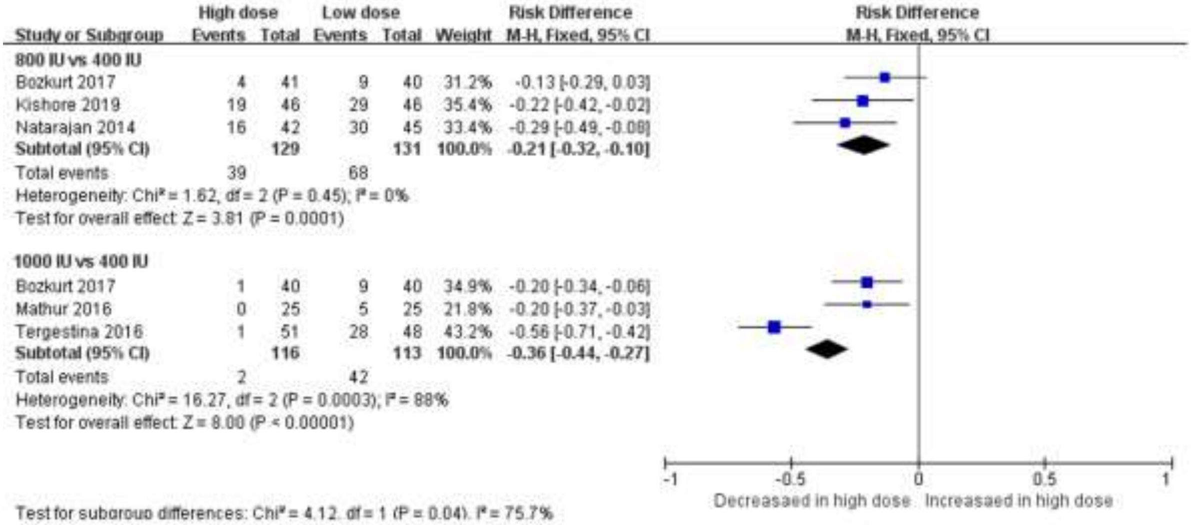

(L) Vitamin D excess according to the different high doses

Study or Subgroup	High dose Events	High dose Total	Low dose Events	Low dose Total	Weight	Risk Difference M-H, Fixed, 95% CI
**800 IU vs 400 IU**						
Abdel-Hady 2019	0	25	0	25	30.7%	0.00 [-0.07, 0.07]
Anderson-Berry 2017	0	16	0	16	19.6%	0.00 [-0.11, 0.11]
Bozkurt 2017	1	41	0	40	49.7%	0.02 [-0.04, 0.09]
**Subtotal (95% CI)**		**82**		**81**	**100.0%**	**0.01 [-0.04, 0.06]**
Total events	1		0			
Heterogeneity: $Chi^2 = 0.28$, df = 2 (P = 0.87); $I^2 = 0\%$						
Test for overall effect: Z = 0.50 (P = 0.62)						
**1000 IU vs 400 IU**						
Bozkurt 2017	1	40	0	40	44.7%	0.03 [-0.04, 0.09]
Tergestina 2016	5	51	0	48	55.3%	0.10 [0.01, 0.19]
**Subtotal (95% CI)**		**91**		**88**	**100.0%**	**0.07 [0.01, 0.12]**
Total events	6		0			
Heterogeneity: $Chi^2 = 1.94$, df = 1 (P = 0.16); $I^2 = 48\%$						
Test for overall effect: Z = 2.20 (P = 0.03)						

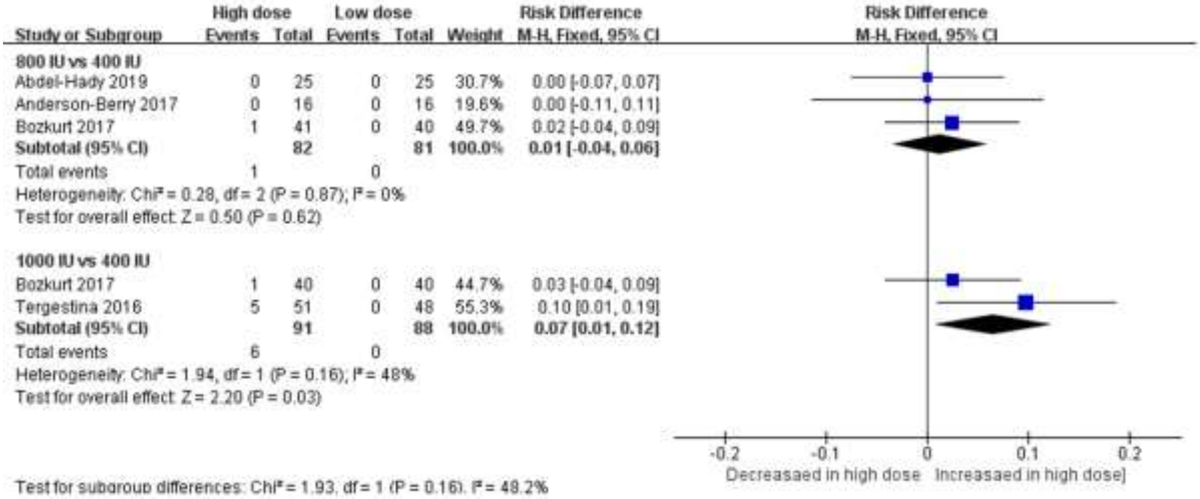

Test for subgroup differences: $Chi^2 = 1.93$, df = 1 (P = 0.16), $I^2 = 48.2\%$
