# Supplementary material for: Short-term and long-term effects of vitamin D supplementation for preterm infants: a systematic review and meta-analysis
Source: J Perinatol. 2025 Oct 7;46(3):425–36. doi: 10.1038/s41372-025-02440-9 (PMC13008753; doi:10.1038/s41372-025-02440-9)
Supplement: Supplementary file 3 — Supplementary Fig. 3 [file 41372_2025_2440_MOESM3_ESM.pdf]

Supplemental Figure 3. Forest plot for each long-term(after 40 weeks' postmenstrual age or from follow up to outpatient clinic after discharge) outcome variable comparing high-dose(≥800IU/day) and low dose(<800IU/day) vitamin D supplementation for preterm infants. Each study identified by first author and year.

(A) Serum 25-hydroxyvitamin D level (ng/mL)

	High dose			Low dose				Mean Difference
Study or Subgroup	Mean	SD	Total	Mean	SD	Total	Weight	IV, Fixed, 95% CI
Backström 1999a	36.14	10.74	16	34.62	9.58	19	75.9%	1.52 [-5.28, 8.32]
Backström 1999b	71.16	35.23	36	68.36	41.37	31	10.2%	2.80 [-15.76, 21.36]
Golan-Tripto 2020	31.25	30.04	13	32.45	29.24	10	5.9%	-1.20 [-25.59, 23.19]
Pittard Iii 1991	49	25	9	51	19	8	8.0%	-2.00 [-22.98, 18.98]
**Total (95% CI)**			**74**			**68**	**100.0%**	**1.21 [-4.72, 7.13]**

Heterogeneity: Chi² = 0.16, df = 3 (P = 0.98); I² = 0%
Test for overall effect: Z = 0.40 (P = 0.69)

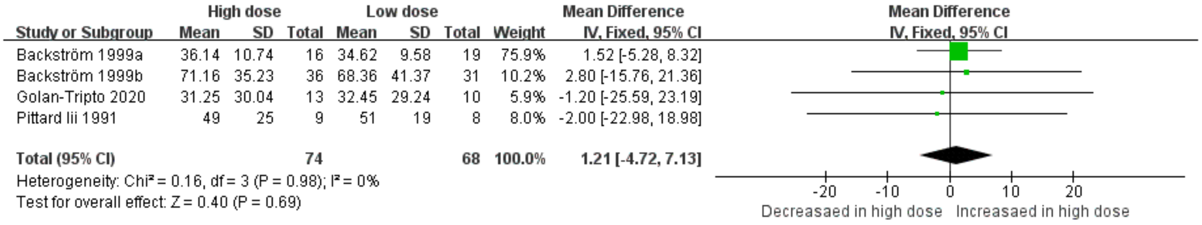

(B) Bone mineral density (mg/cm^2)

	High dose			Low dose				Mean Difference
Study or Subgroup	Mean	SD	Total	Mean	SD	Total	Weight	IV, Fixed, 95% CI
Backström 1999a	113	9	18	109	14	21	63.3%	4.00 [-3.29, 11.29]
Natarajan 2014	152	19	36	158	21	32	36.7%	-6.00 [-15.56, 3.56]
**Total (95% CI)**			**54**			**53**	**100.0%**	**0.33 [-5.47, 6.12]**

Heterogeneity: Chi² = 2.66, df = 1 (P = 0.10); I² = 62%
Test for overall effect: Z = 0.11 (P = 0.91)

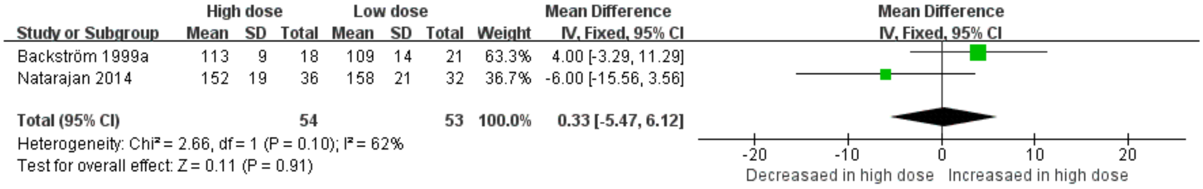

(C) Mortality

	High dose		Low dose			Risk Difference
Study or Subgroup	Events	Total	Events	Total	Weight	M-H, Fixed, 95% CI
Salas 2018	5	30	10	34	100.0%	-0.13 [-0.33, 0.08]
**Total (95% CI)**		**30**		**34**	**100.0%**	**-0.13 [-0.33, 0.08]**
Total events	5		10			

Heterogeneity: Not applicable
Test for overall effect: Z = 1.23 (P = 0.22)

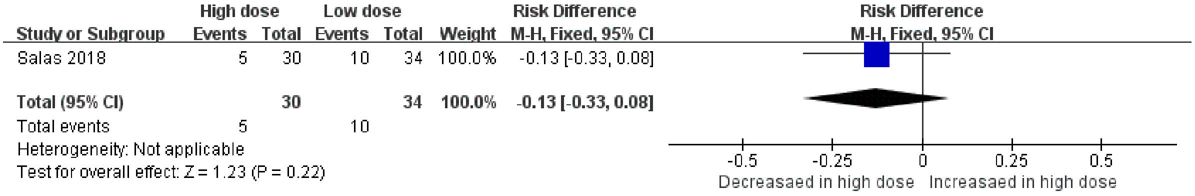

(D) Neurodevelopment

	High dose		Low dose			Risk Difference
Study or Subgroup	Events	Total	Events	Total	Weight	M-H, Fixed, 95% CI
**Cognitive impairment**						
Salas 2018	6	20	8	22	100.0%	-0.06 [-0.35, 0.22]
**Subtotal (95% CI)**		**20**		**22**	**100.0%**	**-0.06 [-0.35, 0.22]**
Total events	6		8			
Heterogeneity: Not applicable						
Test for overall effect: Z = 0.44 (P = 0.66)						
**Language impairment**						
Salas 2018	9	20	12	21	100.0%	-0.12 [-0.43, 0.18]
**Subtotal (95% CI)**		**20**		**21**	**100.0%**	**-0.12 [-0.43, 0.18]**
Total events	9		12			
Heterogeneity: Not applicable						
Test for overall effect: Z = 0.78 (P = 0.43)						
**Neurodevelopmental impairment**						
Salas 2018	6	20	10	23	100.0%	-0.13 [-0.42, 0.15]
**Subtotal (95% CI)**		**20**		**23**	**100.0%**	**-0.13 [-0.42, 0.15]**
Total events	6		10			
Heterogeneity: Not applicable						
Test for overall effect: Z = 0.93 (P = 0.35)						

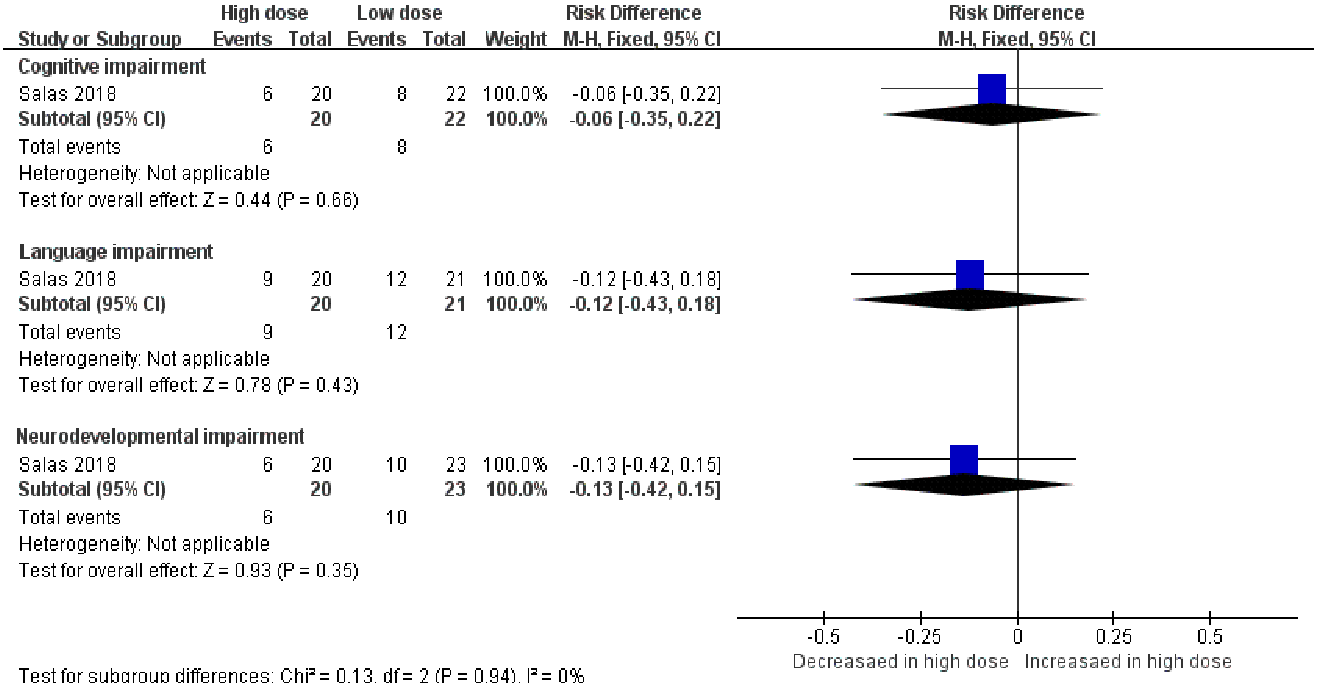

Test for subgroup differences: Chi² = 0.13, df = 2 (P = 0.94), I² = 0%
