# Supplementary material for: Short-term and long-term effects of vitamin D supplementation for preterm infants: a systematic review and meta-analysis
Source: J Perinatol. 2025 Oct 7;46(3):425–36. doi: 10.1038/s41372-025-02440-9 (PMC13008753; doi:10.1038/s41372-025-02440-9)
Supplement: Supplementary file 4 — Supplementary Fig. 4 [file 41372_2025_2440_MOESM4_ESM.pdf]

Supplemental Figure 4. Risk of bias according to the Revised Cochrane risk-of bias (ROB) tool 2 for randomized trials for each study included in the meta analyses assessing the effects of different doses of vitamin D supplementation for preterm infants.

A. Risk of bias graph by domain: review authors' judgements about each risk of bias item presented as percentages across all included studies.

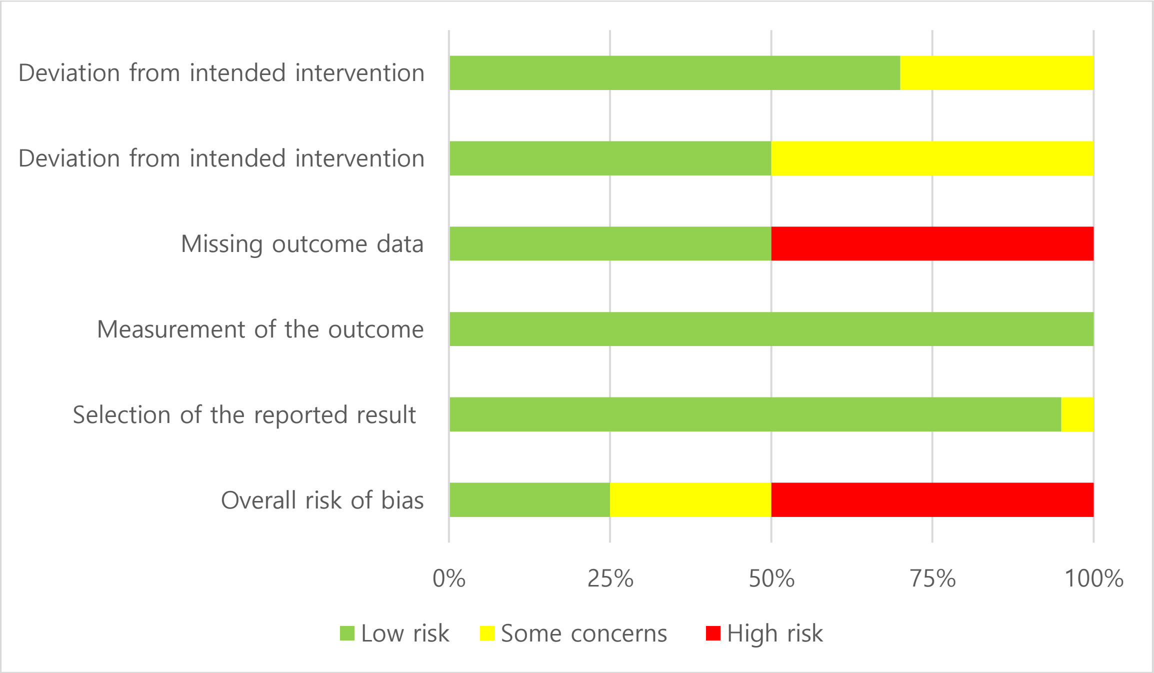

B. Risk of bias graph for individual study : review authors' judgements about each risk of bias item for each included study.

Study (First author, year)	Randomization process	Deviations from the intended interventions	Missing outcome data	Measurement of the outcome	Selection of the reported result	Overall
Abdel-Hady, 2019	Low risk	Low risk	Low risk	Low risk	Low risk	Low risk
Alizade, 2006	Some Concerns	Some Concerns	Low risk	Low risk	Low risk	Some Concerns
Aly, 2019	Low risk	Low risk	Low risk	Low risk	Low risk	Low risk
Anderson-Berry, 2017	Low risk	Low risk	Low risk	Low risk	Low risk	Low risk
Aristizabal, 2022	Some Concerns	Low risk	High risk	Low risk	Low risk	High risk
Backström, 1999a	Low risk	Some Concerns	Low risk	Low risk	Low risk	Some Concerns
Backström, 1999b	Low risk	Some Concerns	Low risk	Low risk	Low risk	Some Concerns
Bozkurt, 2017	Low risk	Some Concerns	High risk	Low risk	Low risk	High risk
Evans, 1989	Some Concerns	Some Concerns	High risk	Low risk	Low risk	High risk
Fort 2016	Low risk	Low risk	High risk	Low risk	Some Concerns	High risk
Golan-Tripto, 2020	Low risk	Low risk	High risk	Low risk	Low risk	High risk
Hanson,2016	Low risk	Low risk	Low risk	Low risk	Low risk	Low risk
Kislal, 2008	Some Concerns	Some Concerns	High risk	Low risk	Low risk	High risk
Koo, 1995	Low risk	Some Concerns	High risk	Low risk	Low risk	High risk
Mathur, 2016	Low risk	Low risk	Low risk	Low risk	Low risk	Low risk
Natarajan, 2014	Low risk	Some Concerns	High risk	Low risk	Low risk	High risk
Pittard, 1991	Some Concerns	Low risk	Low risk	Low risk	Low risk	Some Concerns
Robinson, 1981	Some Concerns	Low risk	Low risk	Low risk	Low risk	Some Concerns
Salas, 2018	Low risk	Some Concerns	High risk	Low risk	Low risk	High risk
Tergestina, 2016	Low risk	Some Concerns	High risk	Low risk	Low risk	High risk

● Low risk ● Some Concerns ● High risk
