# Supplementary material for: Short-term and long-term effects of vitamin D supplementation for preterm infants: a systematic review and meta-analysis
Source: J Perinatol. 2025 Oct 7;46(3):425–36. doi: 10.1038/s41372-025-02440-9 (PMC13008753; doi:10.1038/s41372-025-02440-9)
Supplement: Supplementary file 5 — Supplementary Fig. 5 [file 41372_2025_2440_MOESM5_ESM.pdf]

Supplemental Figure 5. Funnel plot of the meta-analysis of studies that published the effects of high-dose and low-dose vitamin D supplementation on short-term serum 25-hydroxyvitamin D levels

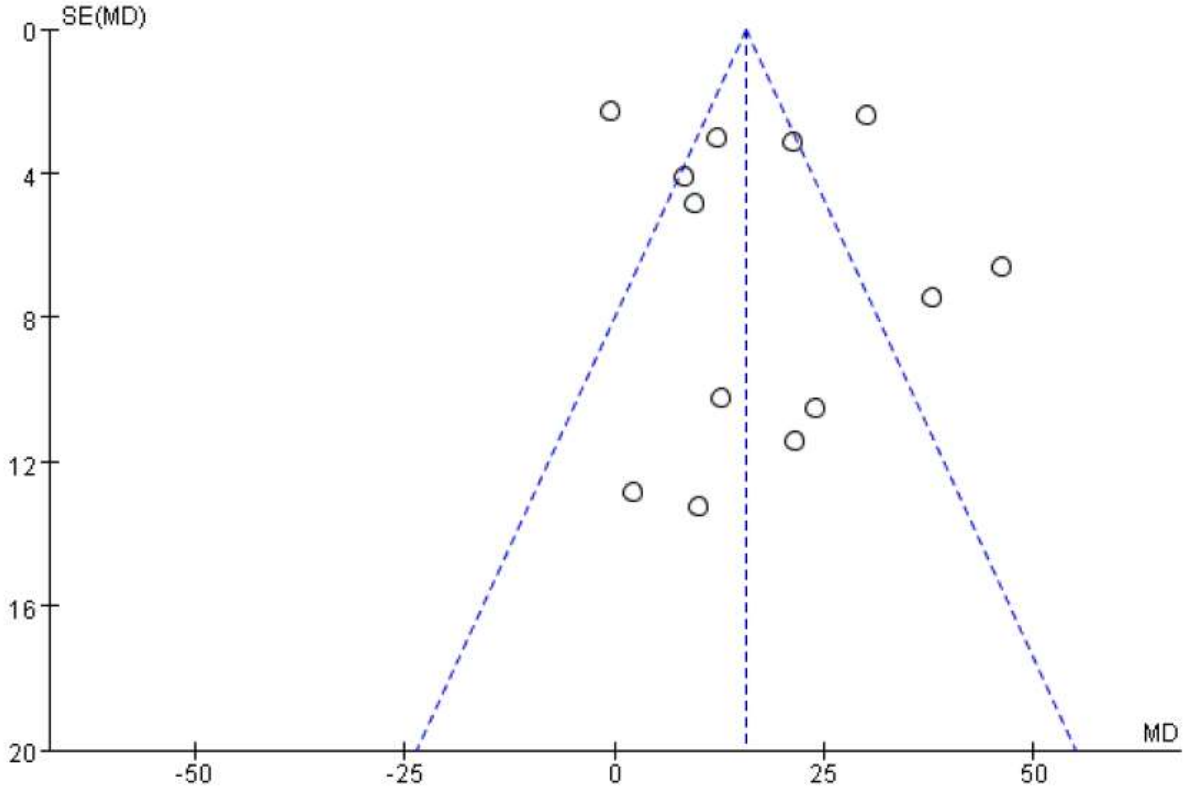

Abbreviations

MD, mean difference; SE, standard error
