# Supplementary material for: Short-term and long-term effects of vitamin D supplementation for preterm infants: a systematic review and meta-analysis
Source: J Perinatol. 2025 Oct 7;46(3):425–36. doi: 10.1038/s41372-025-02440-9 (PMC13008753; doi:10.1038/s41372-025-02440-9)
Supplement: Supplementary file 6 — Supplementary Table 1 [file 41372_2025_2440_MOESM6_ESM.docx]

**Supplemental Table 1. Results of sensitivity analysis regarding the effects of different vitamin D doses conducted according to the risk of bias, funding/conflict of interest status, publication year, and Mentel-Haenszel analysis as Odds ratio**

| **Short-term outcomes** | | |
| --- | --- | --- |
| **Outcome** | **Study (Enrolled study number)** | **Mean difference (95% CI)** |
| Serum 25(OH)D  level (ng/mL) | All (13) ^16,19,21,23-25,27,30-34,36^ | 15.62 (13.35, 17.88) |
|  | High risk studies excluded (7)^16,19,21,27,31,33,34^ | 8.11 (5.07, 11.15) |
|  | Studies with unclear funding or COI excluded (9)^16,19,23,25,27,30,32,33,36^ | 26.78 (23.15, 30.40) |
|  | Feeding clearly described and total vitamin D intake estimated (8)^21,23,25,30-33,36^ | 21.13 (18.31, 23.96) |
|  | Feeding type mentioned, but vitamin D intake not estimated (4)^16,19,24,34^ | 5.02 (0.90, 9.14) |
|  | Studies published since 2010 (8)^16,19,23,25,27,31,32,36^ | 21.55 (18.68, 24.43) |
| Weight gain velocity (g/day) | All (2)^30,31^ | 2.57 (1.10, 4.04) |
|  | High risk studies excluded (1)^31^ | 2.80 (1.00, 4.60) |
|  | Studies with unclear funding or COI excluded (1)^30^ | 2.11 (-0.45, 4.67) |
|  | Studies published since 2010 (1)^31^ | 2.80 (1.00, 4.60) |
| Length gain velocity (cm/week) | All (2)^30,31^ | 1.01 (0.22, 1.80) |
|  | High risk studies excluded (1)^31^ | 1.20 (0.20, 2.20) |
|  | Studies with unclear funding or COI excluded (1)^30^ | 0.70 (-0.58, 1.98) |
|  | Studies published since 2010 (1)^31^ | 1.20 (0.20, 2.20) |
| Head circumference gain velocity (cm/week) | All (2)^30,31^ | 0.57 (0.13, 1.02) |
|  | High risk studies excluded (1)^31^ | 0.50 (-0.07, 1.07) |
|  | Studies with unclear funding or COI excluded (1)^30^ | 0.70 (-0.04, 1.44) |
|  | Studies published since 2010 (1)^31^ | 0.50 (-0.07, 1.07) |
| Length of hospital stay (days) | All (4)^16,18,19,23^ | -2.15 (-4.60, 0.30) |
|  | High risk studies excluded (3)^16,18,19^ | -2.69 (-5.28, -0.11) |
|  | Studies with unclear funding or COI excluded (3)^16,18,19^ | -2.69 (-5.28, -0.11) |
|  | Feeding clearly described and total vitamin D intake estimated (1)^23^ | 2.07 (-5.03. 10.43) |
|  | Feeding type mentioned, but vitamin D intake not estimated (3)^16,18,19^ | -2.69 (-5.28, -0.11) |
|  | Studies published since 2010 (4)^16,18,19,23^ | -2.15 (-4.60, 0.30) |
| Serum PTH (pg/mL) | All (4)^19,23,31,36^ | -15.76 (-21.96, -9.56) |
|  | High risk studies excluded (2)^19,31^ | -15.00 (-21.61, -8.38) |
|  | Studies with unclear funding or COI excluded (2)^19,33^ | -0.62 (-12.92, 11.69) |
|  | Feeding clearly described and total vitamin D intake estimated 8(3)^23,31,36^ | -22.22 (-29.22, -15.21) |
|  | Feeding type mentioned, but vitamin D intake not estimated (1)^19^ | 7.50 (-5.79, 20.79) |
|  | Studies published since 2010 (4)^19,23,31,36^ | -15.76 (-21.96, -9.56) |
| Serum calcium  (mg/dL) | All (9)^16,17,19,23,25,29,30,31,36^ | 0.12 (0.03, 0.21) |
|  | High risk studies excluded (4)^16,17-19^ | 0.17 (0.06, 0.29) |
|  | Studies with unclear funding or COI excluded (5)^16,19,25,30,35^ | 0.06 (-0.08, 0.20) |
|  | Feeding clearly described and total vitamin D intake estimated (6) ^23,25,29,30,31,36^ | 0.25 (0.13, 0.37) |
|  | Feeding type mentioned, but vitamin D intake not estimated (3)^16,17,19^ | -0.06 (-0.23, 0.11) |
|  | Studies published since 2010 (6)^16,19,23,25,31,36^ | 0.19 (0.08, 0.29) |
| Serum phosphorus  (mg/dL) | All (8)^16,17,23,25,29,30,31,36^ | -0.06 (-0.23, 0.11) |
|  | High risk studies excluded (3)^16,17,31^ | 0.20 (-0.05, 0.45) |
|  | Studies with unclear funding or COI excluded (4)^16,25,30,36^ | -0.07 (-0.32, 0.18) |
|  | Feeding clearly described and total vitamin D intake estimated (6)^23,25,29,30,31,36^ | -0.14 (-0.35, 0.06) |
|  | Feeding type mentioned, but vitamin D intake not estimated (2)^16,17^ | 0.11 (-0.20, 0.41) |
|  | Studies published since 2010 (5) ^16,23,25,31,36^ | 0.04 (-0.16, 0.23) |
| Serum ALP (U/L) | All (7)^16,17,23,29-31,36^ | -3.02 (-24.17, 18.13) |
|  | High risk studies excluded (3)^16,17,31^ | -12.94 (-38.51, 12.63) |
|  | Studies with unclear funding or COI excluded (3)^16,30,36^ | -0.36 (-31.27, 30.56) |
|  | Feeding clearly described and total vitamin D intake estimated (5)^23,29,30,31,36^ | -13.93 (-44.66, 16.81) |
|  | Feeding type mentioned, but vitamin D intake not estimated (2)^16,17^ | 6.79 (-22.37, 35.94) |
|  | Studies published since 2010 (4) | -20.10 (-48.55, 8.35) |
| Serum osteocalcin  (ng/mL) | All (3)^24,29,30^ | 1.08 (-0.89, 4.49) |
|  | Studies with unclear funding or COI excluded (2)^24,30^ | 2.01 (-0.78, 4.79) |
|  | Feeding clearly described and total vitamin D intake estimated (2)^29,30^ | 1.06 (-2.96, 5.09) |
|  | Feeding type mentioned, but vitamin D intake not estimated (1)^24^ | 2.40 (-1.22, 6.02) |
|  | Studies published since 2010 (0) |  |
| uCa/Cr | All (3)^16,23,30^ | 0.00 (-0.08, 0.08) |
|  | High risk studies excluded (1)^16^ | 0.00 (-0.09, 0.09) |
|  | Studies with unclear funding or COI excluded (2)^16,30^ | 0.00 (-0.08, 0.09) |
|  | Feeding clearly described and total vitamin D intake estimated (2)^29,30^ | -0.03 (-0.22, 0.17) |
|  | Feeding type mentioned, but vitamin D intake not estimated (1)^16^ | 0.00 (-0.09, 0.09) |
|  | Studies published since 2010 (2)^16,20^ | -0.01 (-0.09, 0.07) |
| **Outcome** | **Study (Enrolled study number)** | **Risk difference (95% CI)** |
| Vitamin D deficiency | All (5)^23,28,30,32,36^ | -0.29 (-0.37, -0.22) |
|  | High risk studies excluded (1)^31^ | -0.20 (-0.34, -0.06) |
|  | Studies with unclear funding or COI excluded (2)^32,36^ | -0.43 (-0.56, -0.31) |
|  | Feeding clearly described and total vitamin D intake estimated (4)^23,31,32,36^ | -0.32 (-0.04, -0.23) |
|  | Feeding type mentioned, but vitamin D intake not estimated (0) | . |
|  | Studies published since 2010 (5)^23,28,31,32,36^ | -0.29 (-0.37, -0.22) |
|  | Mantel-Haenszel, **Odds ratio** (5) ^23,28,31,32,36^ | 0.20 (0.12, 0.32) |
| Vitamin D excess | All (4)^16,19,23,36^ | 0.04 (0.00, 0.08) |
|  | High risk studies excluded (2)^16,19^ | 0.00 (-0.07, 0.07) |
|  | Studies with unclear funding or COI excluded (3)^16,19,36^ | 0.05 (-0.00, 0.11) |
|  | Feeding clearly described and total vitamin D intake estimated (2)^23,36^ | 0.06 (0.01, 0.11) |
|  | Feeding type mentioned, but vitamin D intake not estimated (2)^16,19^ | 0.00 (-0.07, 0.07) |
|  | Studies published since 2010 (4)^16,19,23,36^ | 0.04 (0.00, 0.08) |
|  | Mantel-Haenszel, **Odds ratio** (2)^23,36^ | 6.26 (0.79, 49.39) |
| Skeletal hypomineralization | All (4)^17,19,31,34^ | -0.18 (-0.28, -0.08) |
|  | Studies with unclear funding or COI excluded (1)^19^ | -0.38 (-0.43, -0.32) |
|  | Studies published since 2010 (2)^19,31^ | -0.37 (-0.56, -0.17) |
|  | Feeding clearly described and total vitamin D intake estimated (1)^31^ | -0.36 (-0.42, -0.30) |
|  | Feeding type mentioned, but vitamin D intake not estimated (3)^17,19,34^ | -0.10 (-0.20, -0.01) |
|  | Mantel-Haenszel, **Odds ratio** (2)^19,31^ | 0.20 (0.08, 0.52) |
| Mortality | All (2)^16,25^ | -0.13 (-0.25, -0.02) |
|  | High risk studies excluded (1)^25^ | -0.08 (-0.26, 0.10) |
|  | Feeding clearly described and total vitamin D intake estimated (1)^25^ | -0.17 (-0.35. 0.01) |
|  | Feeding type mentioned, but vitamin D intake not estimated (1)^16^ | -0.08 (-0.26, 0.10) |
|  | Studies published since 2010 (2)^16,25^ | -0.13 (-0.25, -0.02) |
|  | Mantel-Haenszel, **Odds ratio** (2) ^16,25,^ | 0.25 (0.07, 0.96) |
| Respiratory distress syndrome | All (2)^23,25^ | -0.04 (-0.16, 0.09) |
|  | Studies with unclear funding or COI excluded (1)^25^ | -0.04 (-0.23, 0.15) |
|  | Studies published since 2010 (2)^23,25^ | -0.04 (-0.16, 0.09) |
|  | Mantel-Haenszel, **Odds ratio** (2) ^23,25^ | 0.82 (0.41, 1.67) |
| Bronchopulmonary dysplasia | All (5)^16,20,21,23,25^ | -0.02 (-0.11, 0.07) |
|  | High risk studies excluded (2)^16,21^ | -0.01 (-0.12, 0.10) |
|  | Studies with unclear funding or COI excluded (3)^16,20,25^ | -0.01 (-0.13, 0.12) |
|  | Feeding clearly described and total vitamin D intake estimated (4)^20,21,23,25^ | -0.03 (-0.13, 0.08) |
|  | Feeding type mentioned, but vitamin D intake not estimated (1)^16^ | 0.00 (-0.07, 0.07) |
|  | Studies published since 2010 (4) ^16,20,25,23^ | -0.02 (-0.12, 0.08) |
|  | Mantel-Haenszel, **Odds ratio** (4)^20,21,23,25^ | 0.87 (0.50, 1.54) |
| Late onset sepsis | All (2)^23,25^ | 0.01 (-0.10, 0.12) |
|  | Studies with unclear funding or COI excluded (1)^25^ | -0.16 (-0.29, -0.03) |
|  | Studies published since 2010 (2)^23,25^ | 0.01 (-0.10, 0.12) |
|  | Mantel-Haenszel, **Odds ratio** (2)^23,25^ | 1.07 (0.50, 2.29) |
| **Long-term outcomes** | | |
| **Outcome** | **Study (Enrolled study number)** | **Mean difference (95% CI)** |
| Serum 25(OH)D  (ng/mL) | All (4)^21,22,26,33^ | 1.21 (-4.72, 7.13) |
|  | High risk studies excluded (3)^21,22,33^ | 1.36 (-4.75, 7.47) |
|  | Studies with funding source excluded (2)^26,33^ | 1.67 (-4.71, 8.06) |
|  | Feeding clearly described and total vitamin D intake estimated (3)^21,22,33^ | 1.36 (-4.75, 7.47) |
|  | Feeding type mentioned, but vitamin D intake not estimated (1)^26^ | -1.20 (-25.59, 23.19) |
|  | Studies published since 2010 (1)^26^ | -1.20 (-25.59, 23.19) |
| Bone mineral density (mg/cm^2^) | All (2)^21,32^ | 0.33 (-5.47, 6.12) |
|  | High risk studies excluded (1)^21^ | 4.00 (-3.29, 11.29) |
|  | Studies published since 2010 (1)^32^ | -6.00 (-15.56, 3.56) |

Abbreviations

CI, confidence interval; COI, conflict of interest; PTH, parathyroid hormone; uCa/Cr, urine calcium/creatinine ratio; 25(OH)D, 25-hydroxyvitamin D
