# Supplementary material for: Short-term and long-term effects of vitamin D supplementation for preterm infants: a systematic review and meta-analysis
Source: J Perinatol. 2025 Oct 7;46(3):425–36. doi: 10.1038/s41372-025-02440-9 (PMC13008753; doi:10.1038/s41372-025-02440-9)
Supplement: Supplementary file 7 — Supplementary Table 2 [file 41372_2025_2440_MOESM7_ESM.docx]

**Supplemental Table 2. Definitions of outcome measures in each study outcome measures**

| **Short-term outcomes:** the latest follow-up before 40 weeks’ post menstrual age or discharge from neonatal intensive care unit  **Long-term outcomes:** the latest follow-up after 40 weeks’ postmenstrual age or from follow up to outpatient clinic after discharge | |
| --- | --- |
| Vitamin D deficiency | Serum 25(OH)D^a^ level < 20 ng/mL |
| Vitamin D excess | Abdel-Hady 2019: Serum 25(OH)D level > 100 ng/mL  Anderson-Berry 2017: tetany or hypercalcemia  Bozkurt 2017: Serum 25(OH)D level > 100 ng/mL  Tergestina 2016: Serum 25(OH)D level > 70 ng/mL |
| Skeletal hypomineralization | Alizade 2006: osteopenia, bone fractures, intracortical resorption  Anderson-Berry 2017: dual energy x-ray absorptiometry bone density measurements <10 percentile  Mathur 2016: ≥ Grade 1 by Koo’s criteria  Robinson 1981: radiological evidence of rickets from left wrist |
| Respiratory distress syndrome | Bozkurt 2017: not described  Fort 2016: not described |
| Bronchopulmonary dysplasia | Abdel-Hady 2019: not described  Aristizabal 2023: any supplemental oxygen requirement at 36 weeks of postmenstrual age  Backström 1999a: not described  Bozkurt 2017: not described  Fort 2016: 30% FiO2 at 28 days |
| Late-onset sepsis | Bozkurt 2017: not described  Fort 2016: > 72hrs |
| Mortality | Any death |
| Cognitive impairment | Cognitive composite score < 85 from BSID III^b^ |
| Language impairment | Language composite score < 85 from BSID III |
| Neurodevelopmental impairment | Cognitive composite score on the BSID III of less than 85, moderate or severe cerebral palsy with a Gross Motor Function Classification System score of 2 or higher, hearing impairment, or bilateral visual impairment |

^a^25(OH)D = 25-hydroxyvitamin D; to convert 25(OH)D from ng/mL to nmol/L, multiply by 2.5

^b^BSID III = Bayley Scales of Infant and Toddler Development, third edition
